# CO_2_-Induced Transcriptional Reorganization: Molecular Basis of Capnophillic Lactic Fermentation in *Thermotoga neapolitana*

**DOI:** 10.3389/fmicb.2020.00171

**Published:** 2020-02-18

**Authors:** Giuliana d’Ippolito, Simone Landi, Nunzia Esercizio, Mariamichella Lanzilli, Marco Vastano, Laura Dipasquale, Nirakar Pradhan, Angelo Fontana

**Affiliations:** Bio-Organic Chemistry Unit, Institute of Biomolecular Chemistry, Italian National Research Council (CNR), Pozzuoli, Italy

**Keywords:** lactic acid, pyruvate, glycolysis, hydrogenase, thermophilic, RNA-seq

## Abstract

Capnophilic lactic fermentation (CLF) is a novel anaplerotic pathway able to convert sugars to lactic acid (LA) and hydrogen using CO_2_ as carbon enhancer in the hyperthermophilic bacterium *Thermotoga neapolitana*. In order to give further insights into CLF metabolic networks, we investigated the transcriptional modification induced by CO_2_ using a RNA-seq approach. Transcriptomic analysis revealed 1601 differentially expressed genes (DEGs) in an enriched CO_2_ atmosphere over a total of 1938 genes of the *T. neapolitana* genome. Transcription of PFOR and LDH genes belonging to the CLF pathway was up-regulated by CO_2_ together with 6-phosphogluconolactonase (6PGL) and 6-phosphogluconate dehydratase (EDD) of the Entner–Doudoroff (ED) pathway. The transcriptomic study also revealed up-regulation of genes coding for the flavin-based enzymes NADH-dependent reduced ferredoxin:NADP oxidoreductase (NFN) and NAD-ferredoxin oxidoreductase (RNF) that control supply of reduced ferredoxin and NADH and allow energy conservation-based sodium translocation through the cell membrane. These results support the hypothesis that CO_2_ induces rearrangement of the central carbon metabolism together with activation of mechanisms that increase availability of the reducing equivalents that are necessary to sustain CLF. In this view, this study reports a first rationale of the molecular basis of CLF in *T. neapolitana* and provides a list of target genes for the biotechnological implementation of this process.

## Introduction

*Thermotoga neapolitana* is a hyperthermophilic anaerobic bacterium of the order Thermotogales (Belkin et al., 1986). The taxonomic group shares a rod shape and complex outer envelope called toga that surrounds the bacterial cell and forms a periplasmatic space around the poles (Angel et al., 1993). *T. neapolitana* and other sister species are good candidates for the sustainable and efficient conversion of food and agriculture residues to hydrogen (H_2_) by Dark Fermentation (Conners et al., 2006; Manish and Banerjee, 2008; Hallenbeck and Ghosh, 2009; Guo et al., 2010; d’Ippolito et al., 2010; Elleuche et al., 2014; Pradhan et al., 2015, 2016a).

**FIGURE 1:**
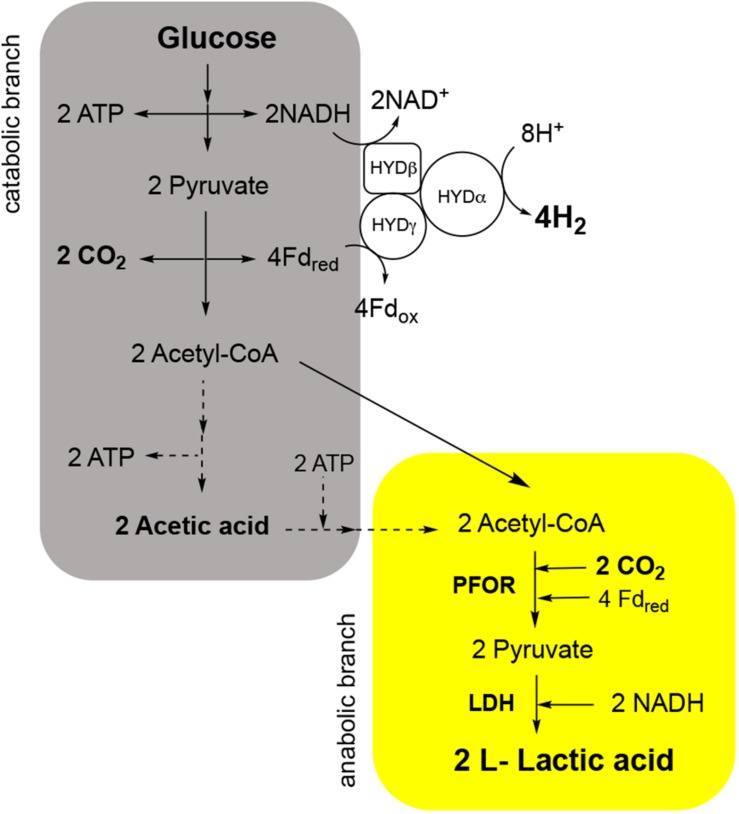
Capnophilic lactic fermentation (CLF) pathway in *T. neapolitana* with details of the enzymatic reactions in the catabolic (gray) and anabolic (yellow) branch in relation to production and use of CO_2._ PFOR, pyruvate:ferredoxin oxidoreductase; LDH, lactate dehydrogenase; HYD, hydrogenase.

In the last years, we reported that *T. neapolitana* also operates a novel, anaplerotic process named capnophilic lactic fermentation (CLF) for the synthesis of almost enantiopure L-lactic acid (LA) without affecting H_2_ production (Dipasquale et al., 2014; d’Ippolito et al., 2014; Pradhan et al., 2016b, 2019; Nuzzo et al., 2019). The metabolic process is activated by CO_2_ (capnophilic means “requiring CO_2_”) and, nominally, is dependent on a Janus pathway including a catabolic branch leading to acetyl-CoA (AcCoA) from sugars by glycolysis, and an anabolic branch that combines AcCoA and CO_2_ to give LA through reduction of newly synthesized pyruvate (PYR) by a NADH-dependent lactic dehydrogenase (LDH) (Figure 1).

This second part of the pathway requires an additional burden of reducing equivalents and determines an unconceivable deviation from Dark Fermentation model for carbon and hydrogen balance (Dipasquale et al., 2014). We showed that CLF is not equally active in all members of the order Thermotogales (Dipasquale et al., 2018) but the almost complete absence of molecular and biochemical studies on metabolism of this group of bacteria has been an insurmountable barrier to explore this process that presumably involves crosstalking of several pathways. The aim of the present work was to investigate the role of CO_2_ as biochemical trigger in *T. neapolitana* and to correlate metabolic change with or without CO_2_ to hydrogen and LA production. We based our analysis on a differential RNA-sequencing of the strain *T. neapolitana* subsp. *capnolactica*, a mutant that shows an incremented operation of CLF (Pradhan et al., 2017). Transcriptome studies were associated to the experimental response of the bacterium to CO_2_. Under our experimental conditions, CO_2_ sparging generates a complex equilibrium between carbon dioxide as a gas or dissolved in the aqueous phase, and its hydrated derivatives H_2_CO_3_, ${\text{HCO}}_{3}^{-}$, ${\text{CO}}_{3}^{=}$. For simplicity we refer to all these chemical forms as CO_2_ throughout the manuscript.

## Materials and Methods

### Biological Material

*Thermotoga neapolitana* subsp. *capnolactica* (DSM 33003) derives from the DSMZ 4359T strain that was stimulated in our laboratory under saturating concentration of CO_2_ (Pradhan et al., 2017). Bacterial cells were grown in a modified ATCC 1977 culture medium containing 10 ml/L of filter-sterilized vitamins and trace element solution (DSM medium 141) together with 10 g/L NaCl, 0.1 g/L KCl, 0.2 g/L MgCl_2_.6H_2_O, 1 g/L NH_4_Cl, 0.3 g/L K_2_HPO_4_, 0.3 g/L KH_2_PO_4_, 0.1 g/L CaCl_2_.2H_2_O, 1 g/L cysteine–HCl, 2 g/L yeast extract, 2 g/L tryptone, 5 g/L glucose, and 0.001 g/L resazurin (d’Ippolito et al., 2010).

### Bacterial Growth

Bacterial precultures (30 mL) were incubated overnight at 80°C without shaking and used to inoculate (6% v/v) cultures in 120 ml serum bottles with a final culture volume of 30 mL. Oxygen was removed by heating until solution was colorless. Cultures were sparged with CO_2_ gas (CLF condition, three bottles) or N_2_ gas (control, three bottles) for 5 min at 30 mL/min. pH was monitored and adjusted to approximately 7.5 by 1 M NaOH. Sparging followed by pH adjustment was repeated every 24 h. Inoculated bottles were maintained in a heater (Binder ED720) at 80°C. Cell growth was determined by optical density (OD) at 540 nm (UV/Vis Spectrophotometer DU 730, Beckman Coulter). Samples (2 ml of medium) were collected from each bottle after 0, 24, and 48 h. After centrifugation at 16,000 × *g* for 15 min (Hermle Z3236K), residues and supernatants were kept at −20°C until analysis. Cell morphology was monitored by microscope observation (Axio VertA1, Carl Zeiss, magnification of 100×).

### Gas Analysis

Gas (H_2_ and CO_2_) measurements were performed by gas chromatography (GC) on an instrument (Focus GC, Thermo Scientific) equipped with a thermoconductivity detector (TCD) and fitted with a 3 m molecular sieve column (Hayesep Q). N_2_ was used as carrier gas. Gas sampling was carried out at 24 and 48 h.

### Chemical Analysis

Glucose concentration was determined by the dinitrosalicylic acid method calibrated on a standard solution of 2 g/L glucose (Bernfeld, 1995). Organic acids were measured by ERETIC ^1^H NMR as described by Nuzzo et al. (2019). All experiments were performed on a Bruker DRX 600 spectrometer equipped with an inverse TCI CryoProbe. Peak integration, ERETIC measurements, and spectrum calibration were obtained by the specific subroutines of Bruker Top-Spin 3.1 program. Spectra were acquired with the following parameters: flip angle = 90°, recycle delay = 20 s, SW = 3000 Hz, SI = 16K, NS = 16, RG = 1. An exponential multiplication (EM) function was applied to the FID for line broadening of 1 Hz. No baseline correction was used.

### RNA Extraction and Sequencing

RNA (three replicates for CLF and three replicates for control) was extracted by standard method with TRIzol (Invitrogen, Carlsbad, CA, United States). Concentration in each sample was determined by a ND-1000 Spectrophotometer (NanoDrop) and quality assessed by Agilent 2100 Bioanalyzer and Agilent RNA 6000 nano kit (Agilent Technologies, Santa Clara, CA, United States). Hi-quality RNA samples were used for high-throughput sequencing. Indexed libraries were prepared from 4 μg/ea purified RNA with TruSeq Stranded mRNA Sample Prep Kit (Illumina) according to the manufacturer’s instructions. Libraries were quantified using the Agilent 2100 Bioanalyzer (Agilent Technologies) and pooled such that each index-tagged sample was present in equimolar amounts, with final concentration of the pooled samples of 2 nM. The pooled samples were subject to cluster generation and sequencing using an Illumina HiSeq 2500 System (Illumina) in a 2 × 100 paired-end format at a final concentration of 8 pmol. RNA extraction and the sequencing service was provided by Genomix4life SRL (Baronissi, Salerno, Italy).

### RNA-Seq Bioinformatic Analysis

Quality check of the sequenced reads was performed using the FAST QC software^1^. The obtained high-quality reads were used for mapping by the Bowtie software (Langmead and Salzberg, 2012). Genome of *T. neapolitana* DSM_4359 at GenBank database (assembly accession: GCA_000018945.1) was used as reference. The quality statistics of the mapping process is showed in the Supplementary Table 1. The counting of the mapped reads was performed by the software HTScount. In order to define the set of expressed genes, raw read counts were normalized using the TMM method (Trimmed mean). Differentially expressed genes (DEGs) were obtained by the DESeq2 package of R language at a false discovery rate (FDR) ≤ 0.05 (Love et al., 2014). Expression values were reported as “Fold Change” (ratio of the normalized expression value in sample over control). Values <1 are shown as (−1/FC) to display negative regulation. Cluster analysis was performed using the MeV software based on the respective normalized reads count values (Howe et al., 2011). The number of clusters was determined by Figure of Merit (FOM) analysis. Clusters were generated by employing *k*-means clustering with Euclidian distances. In gene ontology enrichment analysis (GOEA), categories with a number of entries lower than 4 were excluded in the final output.

### Real-Time PCR

RNAseq results were validated by real-time PCR. Triplicate quantitative assays were performed using a Platinum SYBR Green qPCR SuperMix (Life Technologies, Carlsbad, CA, United States). Cells from control cultures (N_2_ sparging) were used as calibrators and RNA 16S served as endogenous reference gene (Okonkwo et al., 2017). Calculation of gene expression was carried out using the 2^–ΔΔCt^ method as in Livak and Schmittgen (2001). For each sample, mRNA amount was calculated relatively to the calibrator sample for the corresponding genes. Primers used for genes expression analysis are listed in Supplementary Table 2.

### Western Blotting

Bacterial cells were grown under CLF condition and control as described above. Samples were collected after 12, 15, 36, 39, 42, and 45 h. Proteins extraction was performed on 1.5 ml of culture. After centrifugation at 10,000 × *g* for 20 min at 4°C, the pellets were suspended in cracking buffer (100 mM Tris–HCl pH 7.5, 30% glycerol, 2% SDS, 4 mM EDTA, 28 mM β-mercaptoethanol) and incubated at 100°C for 10 min. After centrifugation at 10,000 × *g* at 4°C for 1 h, the supernatants were recovered and protein content was measured by Bradford reagent (Bio-Rad, Hercules, CA, United States). Protein fractionation was performed on SDS polyacrylamide gel using a precast 4–15% Mini-PROTEAN TGX Stain Free (Bio-Rad, Hercules, CA, United States) and electro-transferred on polyvinylidene difluoride membrane (PVDF) using the Trans-Blot Turbo Transfer System (Bio-Rad, Hercules, CA, United States) following the manufacturer’s instructions. The detection of protein was performed by primary antibody raised in rabbit (1:1000; PRIMM Srl, Milan, Italy) against peptide 149–359 of *T. neapolitana* PYR synthase subunit A (PFOR-A) (NCBI database protein accession number YP_002534223.1) and against peptide 1-192 of *T. neapolitana* Fe–Fe hydrogenase subunit β (β-HYD) (NCBI database protein accession number AAC02685.1). Horseradish peroxidase (HRP)-conjugated anti-rabbit secondary antibody was used for staining (Sigma; 1:7000). Western blot filters were visualized by Clarity^TM^ Western ECL substrate (Bio-Rad, Hercules, CA, United States) and subjected to analysis using Image Lab 6.0 Software (Bio-Rad, Hercules, CA, United States). Analysis included the determination of intensity of total Stain-Free fluorescence and intensity of PFOR-A and β-HYD blots for each lane by the “Lane and Bands” tool. Results were expressed as relative percentage of antibody blot intensity respect to intensity of total proteins.

### Data Availability

The raw sequencing data from this study are stored in the NCBI SRA database^2^ and are retrievable under the accession code PRJNA574556.

### Statistics

Each experiment was performed at least in triplicate. Values were expressed as mean ± standard deviation (SD). The statistical significance of OD_540_, qRT-PCR, sugar consumption, organic acid, and H_2_ yields was evaluated through Student’s *t*-test (*p* ≤ 0.05). The differentially expressed data from the RNA-seq approach were filtered using an FDR ≤ 0.05.

## Results

### Differential Response of Bacterial Cell to CO_2_ and N_2_

Sparging of CO_2_ did not affect cell growth (OD_540_) in comparison to control under N_2_ but increased significantly glucose consumption rate. In agreement with previous reports (Dipasquale et al., 2014; Pradhan et al., 2017), the overall effect of CO_2_ on the fermentation process was the increase of LA production without changing H_2_ yield (except for a slight increase at 24 h) in comparison to N_2_-treated cells. This outcome was more evident at 48 h when lactic/acetic acid ratio was 0.23 under N_2_ and 0.39 under CO_2_ (Table 1).

**TABLE 1:** Growth parameters (OD_540_ and percentage of glucose consumption) and product yields (H_2_, acetic acid, and lactic acid – LA) in *T. neapolitana* cultures sparged with N_2_ and CO_2_ after 24 and 48 h.

	N_2_						CO_2_					
	Growth parameters		Yields (mol/mol glucose)				Growth parameters		Yields (mol/mol glucose)			
	OD_540_	Glucose (%)	H_2_	AA	LA	LA/AA	OD_540_	Glucose (%)	H_2_	AA	LA	LA/AA
24 h	1.03 ± 0.01	37 ± 1.6	2.44 ± 0.1	1.16 ± 0.01	0.29 ± 0.01	0.25	1.02 ± 0.01	44 ± 1.6*	2.83 ± 0.2*	1.25 ± 0.06*	0.38 ± 0.02*	0.31
48 h	1.17 ± 0.03	68 ± 5	2.28 ± 0.2	1.11 ± 0.01	0.26 ± 0.01	0.23	1.06 ± 0.05	88 ± 6*	2.70 ± 0.4	1.09 ± 0.02	0.42 ± 0.04*	0.39

Asterisks (*) indicate significant variation between CO_2_ and N_2_ samples at *p*-value ≤ 0.05. AA, acetic acid; LA, lactic acid.

### Massive Molecular Rearrangement Induced by CO_2_ in *T. neapolitana* subsp. *capnolactica*

**FIGURE 2:**
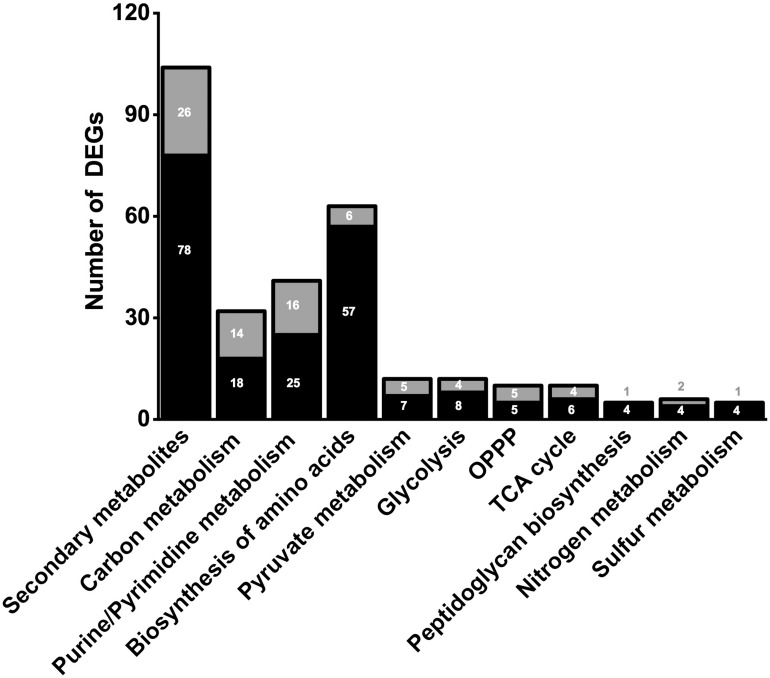
Number of significant (≤0.05 FDR) up-regulated (black) and down-regulated (gray) DEGs of selected metabolic pathways. Number of genes in correlated pathways were identified using KEGG categories.

In order to clarify the molecular effects of CO_2_, an RNA-sequencing approach was performed by using N_2_-treated cells as control. The transcriptomic analysis gave from 19,390,095 to 31,800,340 reads with a percentage of alignment rate >98.59% (Supplementary Table 1). We identified 1601 DEGs between experiments under CO_2_ (CLF samples) and N_2_ (control) (see Supplementary Information). In particular, 612 DEGs showed a significant fold change up to ≥1.5 whereas 593 DEGs were down-regulated (fold change below −1.5) (Figure 2). Considering the whole genome of *T. neapolitana*, CO_2_-dependent DEGs accounted for 83% of the bacterial genes. GOEA of DEGs highlighted change of 45 metabolic networks under CLF conditions, with 26 related to up-regulation and 19 to down-regulation (Supplementary Figure 1). The analysis indicated increased expression of genes related to RNA translation (GO:0006412), ribosome organization (GO:0015934, GO:0015935), transcription factor (TF) (GO:0003700), and amino acids biosynthesis (GO:0009089, GO:0009088, GO:0006526, GO:0009097, GO:0009098, GO:0009073, GO:0006541). Expression and regulation of proteins related to active transport, including major facilitator proteins, and various membrane channels and antiporters, put forward a general down-regulation of sugar transport and vice versa an increased mobilization of phosphate, polyamine, and amino acids. The GOEA also suggested a significant impact of CO_2_ on organization of the cell-wall (GO:0071555) with a marked down-regulation of the degradation of polysaccharides of the outer membrane (xylan catabolic process, GO:0045493; endoglucanase activity, GO:0004519). Interestingly, two of the three genes encoding for the outer membrane proteins that form the structure of toga (Petrus et al., 2012), namely CTN_RS01400 and CTN_RS01395, showed a similar decrease of expression. A number of TFs were also regulated by CO_2_ treatment, including a number of TFs involved in the regulation of sugar catabolism (AraC and LacI). As expected by the simultaneous production of LA and H_2_, we found the enrichment of metabolic categories related to redox reactions (Cell redox homeostasis – GO:0045454; Electron carrier activity – GO:0009055; and Oxidoreductase activity – GO:0016491) and carbon metabolism.

### Central Carbon Metabolism Under CLF Conditions

**FIGURE 3:**
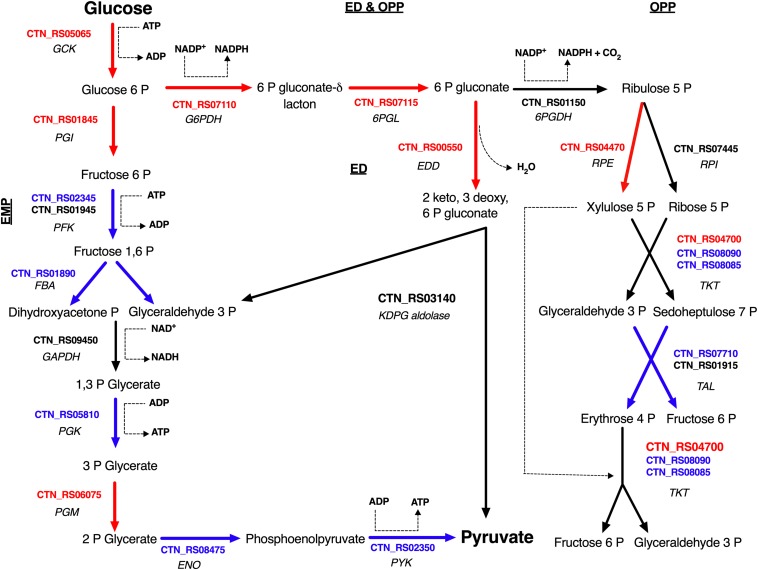
Glucose utilization model in *T. neapolitana* cells under CO_2_. Genes up- and down-regulated are highlighted in red and blue, respectively; NDE genes with 1.5 < fc < −1.5 are in black. GCK, glucokinase; PGI, glucose-6-phosphate isomerase; PFK, 6-phosphofructokinase; FBA, fructose-bisphosphate aldolase; GAPDH, glyceraldehyde-3-phosphate dehydrogenase; PGK, glycerate kinase; PGM, phosphoglycerate mutase; ENO, enolase (Aka phosphoenolate hydratase); PYK, pyruvate kinase; G6PDH, glucose-6-phosphate dehydrogenase; 6PGL, 6-phosphogluconolactonase; EDD, 6-phophoglucono dehydratase; KDPG aldolase, 2-dehydro-3-deoxyphosphogluconate aldolase; 6PGDH, 6-phosphogluconate dehydrogenase; RPE, ribulose 5 phosphate epimerase; RPI, ribulose 5 phosphate isomerase; TKT, transketolase; TAL, transaldolase.

Sparging by CO_2_ induced up-regulation of glucokinase (GCK – CTN_RS05065) and glucose 6-phosphate isomerase (PGI – CTN_RS01845) that catalyze the starting steps of Embden–Meyerhof–Parnas glycolysis (EMP) (Table 2). On the other hand, we found that other enzymes of EMP were significantly down-regulated. Inspection of other genes related to central carbon metabolism revealed a significant transcription of glucose-6-phosphate dehydrogenase (G6PDH – CTN_RS07110) and 6-phosphogluconolactonase (6PGL – CTN_RS07115). These enzymes preside over the synthesis of 6-phospho gluconate that is the first metabolite of the main alternative pathways of glucose catabolism, namely ED and oxidative pentose phosphate (OPP) pathway. On the whole, these data suggest a diversion of carbon flux from EMP to ED or OPP pathways (Figure 3). This evidence was further validated by RT-PCR that showed up-regulation of 6-phosphogluconate dehydratase (EDD – CTN_RS00550) that control the key step of ED and down-regulation of 6-phosphofructokinase (PFK – CTN_RS02345) that is the regulator enzyme of EMP (Supplementary Figure 2). Analysis of the genes of Krebs cycle reveals that *T. neapolitana* apparently lacks the whole pathway. However, we found a significant upregulation of the expression of citrate synthase (CTN_RS01940) and isocitrate dehydrogenase (CTN_RS07145) (Table 2).

**TABLE 2:** List of DEGs related to carbon metabolism.

Locus	Fold change	Description
**Glycolysis – Embden–Meyerhof–Parnas**		
CTN_RS05065	2.18	*Glucokinase*
CTN_RS01845	1.84	*Glucose-6-phosphate isomerase*
CTN_RS02345	–1.79	*6-Phosphofructokinase*
CTN_RS01945	–1.39	*6-Phosphofructokinase. pyrophosphate-dependent*
CTN_RS01890	–1.66	*Fructose-bisphosphate aldolase*
CTN_RS09450	–1.40	*Glyceraldehyde-3-phosphate dehydrogenase*
CTN_RS05810	–1.98	*Glycerate kinase*
CTN_RS06075	1.88	*Phosphoglycerate mutase*
CTN_RS08475	–2.40	*Phosphopyruvate hydratase aka Enolase*
CTN_RS02350	–1.80	*Pyruvate kinase*
**OPP and Entner–Doudoroff common enzymes**		
CTN_RS07110	4.84	*Glucose-6-phosphate 1-dehydrogenase*
CTN_RS07115	5.50	*6-Phosphogluconolactonase*
**Entner–Doudoroff**		
CTN_RS00550	5.83	*6-Phosphogluconate dehydratase*^a^
CTN_RS03140	1.26	*2-Dehydro-3-deoxyphosphogluconate aldolase*
**OPP**		
CTN_RS01150	–1.40	*6-Phosphogluconate dehydrogenase. Decarboxylating*
CTN_RS04470	1.64	*Ribulose-phosphate-epimerase*
CTN_RS07445	NDE	Ribose-5-phosphate isomerase
CTN_RS04700	1.81	Transketolase
CTN_RS08090	–1.60	Transketolase. C-terminal subunit
CTN_RS08085	–2.66	Transketolase. N-terminal subunit
CTN_RS07710	–5.74	Fructose-6-phosphate aldolase
CTN_RS01915	NDE	Fructose-6-phosphate aldolase
**Other genes**		
CTN_RS01940	6.16	*Citrate synthase*
CTN_RS07145	6.35	*Isocitrate dehydrogenase*
CTN_RS09470	2.92	*Fumarate hydratase class I*
CTN_RS00600	–1.81	*Fumarate hydratase. C-terminal subunit*
CTN_RS00605	–2.54	*Fumarate hydratase. N-terminal subunit*
CTN_RS01300	–2.01	*2-Oxoglutarate ferredoxin oxidoreductase. beta subunit*
CTN_RS00595	1.26	*Malate oxidoreductase*

*^*a*^6-Phosphogluconate dehydratase was previously annotated as dihydroxy-acid dehydratase. The correct annotation was performed using a BlastP approach on the Ensamble bacteria database.*

### Regulation of CLF Pathway

**FIGURE 4:**
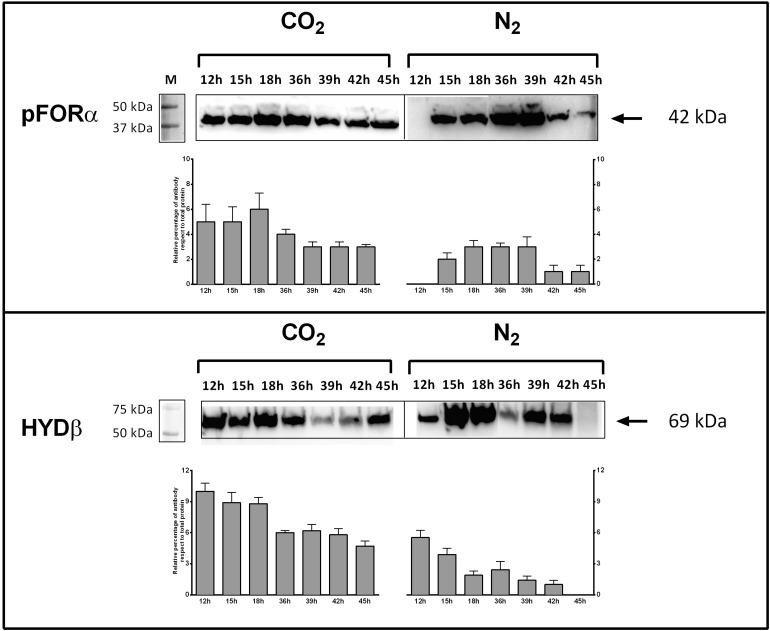
Expression of pyruvate:ferredoxin oxidoreductase (PFOR) and Fe–Fe hydrogenase (HYD) in *T. neapolitana* subsp. *capnolactica*. Bacterial cultures were grown under N_2_ or CO_2_, and collected at 12, 15, 18, 36, 39, 42, and 45 h. PFOR levels were detected by using a polyclonal antibody raised in rabbits against peptide 149–359 of subunit α. HYD levels were detected by using a polyclonal antibody raised in rabbits against peptide 1–192 of subunit β. Western blot filters were subjected to analysis using Image Lab 6.0 Software (Bio-Rad, Hercules, CA, United States). Analysis included the intensity determination of total Stain-Free fluorescence and intensity of PFOR-A and β-HYD blots for each lane. Results were expressed as relative percentage of antibody blot intensity respect intensity of total proteins.

Genes encoding key enzymes of the anabolic branch of CLF pathway were differential expressed by CO_2_ (Table 3). Pyruvate:ferredoxin oxidoreductase (PFOR, E.C. 1.2.7.1) is a heterotetramer enzyme that operates the reversible coupling of AcCoA and CO_2_ to PYR under CLF conditions (d’Ippolito et al., 2014). Each subunit of this enzyme (CTN_RS03380, CTN_RS03385, CTN_RS03390, and CTN_RS03395) showed a significant increase of expression. In consideration of the double function of PFOR as catabolic (from PYR to AcCoA) and anabolic (from AcCoA and CO_2_ to PYR) enzyme, the up-regulation is in good agreement with both the general acceleration of metabolism induced by CO_2_ and the enhanced demand of PYR to feed the downfield synthesis of LA. According to this view, LDH (E.C. 1.1.1.27 – CTN_RS03950) was also up-regulated under the experimental conditions. A specific biochemical character of CLF is the increase of the rate of H_2_ synthesis during the fermentation process (Dipasquale et al., 2014). The molecular analysis revealed a significant up-regulation of the expression of the α-subunit of the bacterial hydrogenase (CTN_RS05285) together with two of the three proteins (CTN_RS06525, HYD E; CTN_RS06535, HYD G) that are necessary to the complex process of maturation of this enzyme. The α-subunit (HYDα) contains the catalytic H-cluster (Schut and Adams, 2009) that is committed to reduction of H^+^ to hydrogen gas. In their model of the hydrogenase of *Thermotoga maritima*, Schut and Adams (2009) have discussed a trimeric bifurcating enzyme that catalyzes multiple electron transfer events simultaneously. HYDα is the final collector of this transfer of electrons from NADH and reduced ferredoxin; thus, it is plausible that the experimentally observed boost of the H_2_ production rate requires an accelerated turnover of this protein under CLF conditions. The CO_2_-induced upregulation of PFOR and HYD was further confirmed by western blotting analysis carried out on PFORα and the β-subunit of hydrogenase (HYDβ) (Figure 4).

**TABLE 3:** List of DEGs related to CLF pathway.

Locus	Fold change	Descriptions
**CFL core enzymes**		
CTN_RS03385	3.07	*Pyruvate synthase subunit porA* (*aka PFOR*α)
CTN_RS03380	1.70	*Pyruvate synthase subunit porB* (*aka PFOR*β)
CTN_RS03395	2.61	*Pyruvate synthase subunit porC* (*aka PFOR*γ)
CTN_RS03390	2.99	*Pyruvate synthase subunit porD* (*aka PFOR*δ)
CTN_RS03950	2.74	*L-lactate dehydrogenase*
CTN_RS05285	1.78	*Fe-hydrogenase alpha subunit*
CTN_RS05290	1.27	*Fe-hydrogenase beta subunit*
CTN_RS05295	NDE	*Fe-hydrogenase gamma subunit*
CTN_RS02020	1.16	*Acetate kinase*
CTN_RS07210	1.78	*Phosphate acetyltransferase*
**Putative energy sustaining enzymes**		
CTN_RS04025	1.50	*NFN* (*NADH-dependent Reduced Ferredoxin:NADP Oxidoreductase*)*^*a*^*
CTN_RS04020	1.77	*NFN*(*NADH-dependent Reduced Ferredoxin:NADP Oxidoreductase*)*^*a*^*
CTN_RS02150	1.39	*Electron transport complex*, *RnfABCDGE type*, *A subunit*
CTN_RS02165	2.67	*Electron transport complex*, *RnfABCDGE type*, *D subunit precursor*
CTN_RS02155	1.93	*Electron transport complex*, *RnfABCDGE type*, *E subunit*
CTN_RS02160	2.52	*Electron transport complex*, *RnfABCDGE type*, *G subunit precursor*
CTN_RS05860	5.96	*Thioredoxin*
CTN_RS08515	3.09	*Thioredoxin reductase*
**Hydrogenase maturation enzymes**		
CTN_RS06540	5.98	*Iron-only hydrogenase system regulator*
CTN_RS06525	7.52	FeFe hydrogenase H-cluster radical SAM maturase HYDE*
CTN_RS01115	1.28	FeFe hydrogenase H-cluster radical SAM maturase HYDF*
CTN_RS06535	6.39	*FeFe hydrogenase H-cluster radical SAM maturase HYDG*
**CoA-related enzyme**		
CTN_RS08445	2.27	*Pantothenate kinase*
**PFOR cofactors**		
CTN_RS08225	5.96	*Ferredoxin*
CTN_RS07010	1.96	*Ferredoxin*
CTN_RS03680	2.55	*Ferredoxin family protein*

^*a*^NADH-dependent reduced ferredoxin:NADP oxidoreductase were previously and uncorrected annotated as dihydroorotate dehydrogenase (CTN_RS04025) and glutamate synthase, beta subunit (CTN_RS04020). The correct annotations were performed using the structures and the sequencing of the *Thermotoga maritima* orthologs (Demmer et al., 2015). *FeFe hydrogenase H-cluster radical SAM maturase HYDEF were previously and uncorrected annotated as biotin synthetase and small GTP binding protein. The correct annotations were performed using a BLASTp approach.

### Redox Re-organization Under CLF Conditions

Enhancement of cell reductants, e.g., NAD(P)H or ferredoxin (Fd), represents a major requirement to sustain the simultaneous production of H_2_ and LA during CLF (Dipasquale et al., 2014; d’Ippolito et al., 2014). RNA-seq approach revealed up-regulation of several genes involved in redox reactions and electron transport. Intriguingly, among up-regulated DEGs we found a significant expression of genes coding for enzymes related to the flavin-based oxidoreductase enzymes NADH-dependent reduced ferredoxin:NADP oxidoreductase (NFN) (CTN_RS04025 and CTN_RS04020) and NAD-Ferredoxin oxidoreductase (RNF) (CTN_RS02165, CTN_RS02160, and CTN_RS02155). Both these proteins are energy conservation systems. NFN controls supply of reduced ferredoxin and NADH by oxidation of NADPH while RNF couples reversible consumption of NADH with synthesis of reduced Fd and trans-membrane transport of Na^+^ or H^+^ (Hess et al., 2013). K-means cluster analysis of DEGs identified five different groups of genes with similar expression profile (not shown). One of the up-regulated cluster (Supplementary Figure 3) included genes coding for PFOR (CTN_RS03385, CTN_RS03390, and CTN_RS03395), flavin-based protein complexes (RNF and NFN – CTN_RS02160, CTN_RS02165, CTN_RS04020, and CTN_RS04025), thioredoxin (CTN_RS05860 and CTN_RS08515), ferredoxin (CTN_RS08225), Na/P cotransporter family protein (CTN_RS07190), and G6PDH (CTN_RS07110). The clustering was coherent with the fermentation results and suggested a CO_2_-stimulated rearrangement of the bacterial metabolism that links NAD(P)H from ED/OPP, PYR synthesis by PFOR, and production of NADH by flavin-based energy conservation systems. According to the experimental results, up-regulation of these pathways could be consistent with increase of the availability of redox potential for the synthesis of LA and H_2_ during CLF.

## Discussion

Thermophilic bacteria, such as *Thermotogales*, have been acquiring an emerging attention in biotechnology for hydrogen production by digestion of organic residues (Conners et al., 2006; Elleuche et al., 2014; Pradhan et al., 2015). Among thermophilic bacteria, *T. neapolitana* shows an interesting potential for the simultaneous production of hydrogen and LA by CLF (Dipasquale et al., 2014; d’Ippolito et al., 2014; Pradhan et al., 2017). In the present study, a selected strain of this bacterium, namely *T. neapolitana* subsp. *capnolactica* (Pradhan et al., 2017) was characterized by comparative analysis of gene expression profiling under CO_2_ (CLF conditions) and N_2_ (control). The study showed transcriptional changes that well correlate with the promoting effect of CO_2_ on both the synthesis of LA and the rate of hydrogen production and glucose consumption. The appearance of this bacterial phenotype was accompanied by variation in the expression of 1601 genes related to central biochemical pathways including glycolysis, TCA, cell wall construction, and protein synthesis. Under light microscopy, the cells showed a typical rod shape with a more homogeneous length than the control bacteria under N_2_ that vice versa were round-shaped (Supplementary Figure 4). In agreement with the acceleration of the fermentation rate, CO_2_ also triggered modification of the expression of 105 genes related to control of extracellular transport. Latif et al. (2015) have recently underlined that adaptation of *Thermotoga maritima* to achieve improved growth fitness requires plasticity of the ABC transporters. We found that expression of several ABC transporters of *T. neapolitana* was differently regulated (Supplementary Information), which is consistent with the modulation of these proteins in response to the different environmental conditions.

These molecular changes were driven by a number of key TFs that were differentially expressed under CO_2_. In particular, we observed different regulation of genes of the LacI family, such as LacI, AraC, and XylR, that are tightly linked to distinct regulons of carbohydrate catabolism (Ravcheev et al., 2014). These proteins have been also identified as regulators of sugar metabolism in Thermotogales, including *T. neapolitana* and *T. maritima* (Rodionov et al., 2013). Thus, control of their expression seems to corroborate the observation that the metabolic acceleration triggered by CO_2_ is sustained by a change of sugar catabolism in association with a more effective import/export of substrates.

According to this view, under CLF conditions, the bacterial cells showed an interesting shift of glucose utilization through down-regulation of EMP and activation of the alternative ED and/or OPP (Figure 3). EMP is energetically more effective than other glycolytic pathways because of the higher ATP yields per glucose unit. However, the energetic cost for the synthesis of the proteins carrying out the 10 reactions of this pathway may represent a growth-limiting factor (Molenaar et al., 2009; Flamholz et al., 2013). Recently, Singh et al. (2018) have discussed the role of OPP in providing additional reductants for H_2_ production in *T. maritima*. ED and OPP show a number of energetic advantages per glucose unit [reduced protein cost or increased amount of NAD(P)H, respectively] in comparison to EMP, thus switch from EMP to ED or OPP can be functional to the increased demand of reducing equivalents during CLF.

**FIGURE 5:**
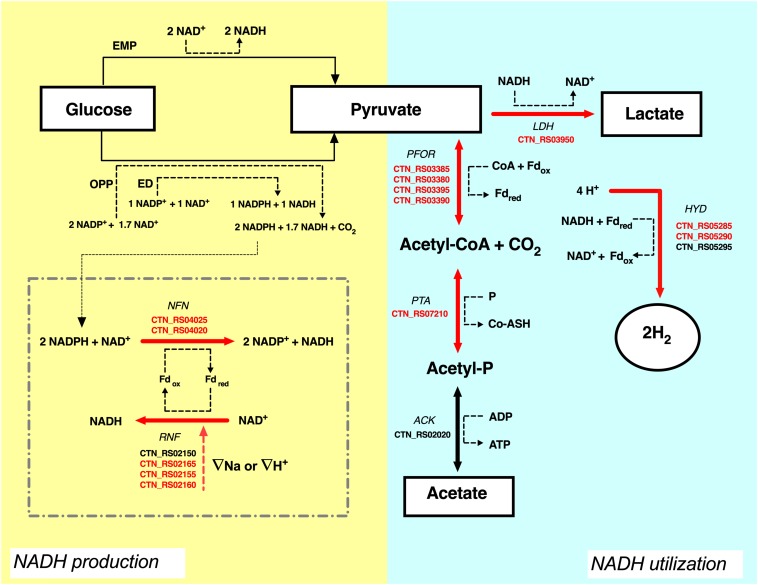
Proposal of carbon and NADH flow in *T. neapolitana* under CO_2_. Electron transfer form glucose (donor) to lactate and hydrogen (final acceptors) is shown. Suggested role of NFN-RNF cycle is shown in the dotted square. Up-regulated genes (≥1.5-fold change) are highlighted in red, NDE genes with 1.5 < fc < −1.5 are in black. ATP is omitted. For explanations see text. ACK, acetate kinase; PTA, phospshotransacetylase; PFOR, pyruvate:ferredoxin oxidoreductase; LDH, lactate dehydrogenase; HYD, hydrogenase; RNF, NAD-ferredoxin oxidoreductase; NFN, NADH-dependent reduced ferredoxin:NADP oxidoreductase; Fd, ferredoxin; EMP, Embden–Meyerhof–Parnas pathway; ED, Entner–Doudoroff pathway; OPP, oxidative pentose phosphate pathway.

The analysis of the transcripts suggested that another pool of NADH could derive from the flavin-based oxido-reductase enzymes NFN and RNF that are both up-regulated by CO_2_. In *T. maritima*, NFN is a bifurcating enzyme that couples the oxidation of NADPH to the exergonic reduction of NAD^+^ and the endergonic reduction of Fd (Demmer et al., 2015). Electron bifurcation is one the mechanisms of energy conservation that biological organisms use to minimize free-energy loss in redox reactions. Bifurcating enzymes catalyze endoergonic and exoergonic electron transfer reactions to circumvent thermodynamic barriers and to carry out reactions that have unfavorable free energy variations (Buckel and Thauer, 2018). Deletion of the NFN complex (Tsac_2085 NfnA and Tsac_2086 NfnB) genes led to the total loss of NADPH-dependent activity and reduces ethanol fermentation in the thermophilic anaerobe *Thermoanaerobacterium saccharolyticum* (Lo et al., 2015). On the other hand, the RNF complex couples the reversible flow of electrons from reduced ferredoxin to NAD^+^. The process is associated with the transport of Na^+^ across the cell membrane and the direction of the electron flow is dependent on the sodium gradient (Biegel et al., 2011; Buckel and Thauer, 2013; Hess et al., 2013). The high production of hydrogen in *T. neapolitana* is associated to consumption of hydrogen ions (H^+^). RNF activity could be related to Na^+^ or H^+^ transport to balance the loss of H^+^ and to maintain the ionic homeostasis in the cell. As suggested in Figure 5, the combination of RNF with NFN may generate a cyclic process leading to production of NADH. In this view, synthesis of LA could be another mechanism to get rid of the excess of reducing equivalents. A similar cycle has been postulated between RNF and ferredoxin-dependent NADH oxidoreductase (FNOR) in *T. saccharolyticum* in order to operate a net transfer of protons across the cell membrane and eliminate the proton gradient that could be lethal for the cells (Tian et al., 2016).

As already reported by Krebs (1941), CO_2_ affects carboxylation reactions also in heterotrophs. In *T. neapolitana*, in addition to the indirect effect on the bacterial metabolism by gene regulation, CO_2_ plays a direct role as chemical reagent in the reaction to give PYR from acetate by PFOR. Thus, increase of the concentration of CO_2_ is very likely the driven force to reverse the oxidative decarboxylation of PYR and to yield carbon fixation. The occurrence of this reaction could explain the experimentally observed presence of PFOR even after the end of sugar consumption by glycolysis (d’Ippolito et al., 2014). At the moment we do not know whether this occurs for inversion of the functionality of PFOR or it is due to a newly synthesized pool of the enzyme (Figure 1). Synthetic PFOR is the key enzyme of the autotrophic fixation by the Wood–Ljungdahl pathway in acetogens (Furdui and Ragsdale, 2000). After our publication of the synthetic activity of PFOR in *T. neapolitana* (d’Ippolito et al., 2014), Xiong et al. (2016) have reported a similar mechanism in *Clostridium thermocellum*. In this work, the authors report assimilation of CO_2_ during cellobiose metabolism with fixation of over 40% of labeled carbon in PYR that derives from PFOR-dependent synthesis. This process is very similar to that of *T. neapolitana* thus suggesting that the activation of the anaplerotic reductive C1 pathway dependent on PFOR can be more common than it is generally believed. It is obvious that the energy requirements of this process need to be considered within a comprehensive organization of the cellular redox pathways.

## Conclusion

In conclusion, the present work showed a correlation between the gene expression, the metabolic adaptation and the biochemical phenotype of the bacterium *T. neapolitana* under conditions that promote CLF. In according with acceleration of H_2_ synthesis, the CO_2_-induced effects clearly indicated an increased energetic metabolism of *T. neapolitana* that is sustained by a concerted reorganization of the cell metabolism including at least variation of central carbon metabolism, activation of anaplerotic oxidoreductase processes and increase of the exchange across the membrane. Similar effects on enhancement of the energetic metabolism or cell growth upon exposure to CO_2_ have been reported in other anaerobic bacteria, such as *Streptococcus thermophilus* (Arioli et al., 2009) and *C. thermocellum* (Xiong et al., 2016). The most significant trait of the molecular adaptation induced by CO_2_ in *T. neapolitana* is related to additional production of reductants by the EMP/ED-OPP switch and the bifurcating mechanisms based on the overexpression of the flavin-based complexes, such as NFN and RFN.

## Data Availability Statement

The raw sequencing data from this study are stored in the NCBI SRA database (http://www.ncbi.nlm.nih.gov) and are retrievable under the accession code PRJNA574556.

## Author Contributions

Gd’I conceived the whole research project, and wrote and amended the manuscript. SL conceived the idea of writing the manuscript, bioinformatic analysis, and wrote and amended the manuscript. NE performed the microbiology and western blot analysis. ML and MV performed the western blot. LD and NP performed the microbiology and sequencing analysis. AF planned and coordinated the whole research project and the experiments along with Gd’I, and wrote and amended the manuscript.

## Conflict of Interest

The authors declare that the research was conducted in the absence of any commercial or financial relationships that could be construed as a potential conflict of interest.

## Footnotes

1 http://www.bioinformatics.babraham.ac.uk/projects/fastqc/

2 http://www.ncbi.nlm.nih.gov
