# Supplementary material for: CO2-Induced Transcriptional Reorganization: Molecular Basis of Capnophillic Lactic Fermentation in Thermotoga neapolitana
Source: Front Microbiol. 2020 Feb 18;11:171. doi: 10.3389/fmicb.2020.00171 (PMC7039931; doi:10.3389/fmicb.2020.00171)
Supplement: Supplementary file 1 [file Data_Sheet_1.pdf]

List of DEGs from *T.neapolitan* subjected to an enriched CO2-atmosphere compared to control, obtained by an RNA seq. Up- (≥ 1,5) and down-regulated genes (≤ 1,5) are highlighted in red and green, respectively.

ID	Fold_Change	Description
CTN_RS00260	26,887	*DUF (domain with unknowk function)*
CTN_RS03985	26,809	*Cold-shock DNA-binding protein family*
CTN_RS00505	23,243	*Ferritin, Dps family protein*
CTN_RS07610	21,327	*ATP phosphoribosyltransferase regulatory subunit*
CTN_RS06555	21,313	*Binding-protein-dependent transport systems inner membrane component precursor*
CTN_RS06240	21,180	*CTN_trnaMet1*
CTN_RS08140	20,956	*Glutamine synthetase*
CTN_RS08775	19,567	*Lipopolysaccharide biosynthesis protein*
CTN_RS04295	19,505	*Cold shock-like protein*
CTN_RS05985	19,235	*CTN_trnaLeu5*
CTN_RS08835	19,027	*peroxiredoxin*
CTN_RS08780	18,866	*CTN_trnaLeu3*
CTN_RS08875	17,039	*BioY protein*
CTN_RS01090	16,961	*CTN_trnaMet2*
CTN_RS08870	16,459	*2-nitropropane dioxygenase, NPD*
CTN_RS06455	16,234	*5-methyltetrahydropteroyltriglutamate--homocysteine methyltransferase*
CTN_RS06560	15,115	*Phosphate ABC transporter, inner membrane subunit PstA*
CTN_RS05450	14,615	*CTN_trnaPro2*
CTN_RS04690	14,499	*CTN_trnaAsp1*
CTN_RS01310	14,158	*Nitrogen regulatory protein P-II*
CTN_RS08865	14,092	*(3R)-hydroxymyristoyl-(acyl carrier protein) dehydratase*
CTN_RS06550	13,643	*Phosphate ABC transporter, periplasmic phosphate-binding protein*
CTN_RS01315	13,365	*Ammonium transporter*
CTN_RS07615	12,399	*ATP phosphoribosyltransferase*
CTN_RS02695	12,314	*Putative uncharacterized protein*
CTN_RS08860	12,236	*3-oxoacyl-(Acyl carrier protein) synthase II*
CTN_RS07250	12,048	*Transcriptional regulator, SARP family*
CTN_RS04795	12,015	*Aspartate-semialdehyde dehydrogenase*
CTN_RS07790	12,003	*Putative uncharacterized protein precursor*
CTN_RS06940	11,567	*CTN_trnaAsn1*

CTN_RS00560	10,768	*Acetolactate synthase, small subunit*
CTN_RS08275	10,658	*Phosphate permease*
CTN_RS08880	10,595	*Malonyl CoA-acyl carrier protein transacylase*
CTN_RS00910	10,464	*CTN_trnaLeu1*
CTN_RS08030	10,448	*1-(5-phosphoribosyl)-5-amino-4-imidazole-carboxylate (AIR) carboxylase*
CTN_RS05465	10,167	*Putative uncharacterized protein precursor*
CTN_RS08035	10,122	*Hypothetical Protein*
CTN_RS02405	9,833	*PP-loop domain protein*
CTN_RS07140	9,528	*Binding-protein-dependent transport systems inner membrane component*
CTN_RS07795	9,204	*Putative uncharacterized protein precursor*
CTN_RS08025	8,840	*PP-loop domain protein*
CTN_RS04800	8,811	*Diaminopimelate epimerase*
CTN_RS02555	8,744	*CTN_trnaAla2*
CTN_RS07125	8,670	*Oligopeptide/dipeptide ABC transporter, ATPase subunit*
CTN_RS07120	8,595	*Oligopeptide ABC transporter, permease protein*
CTN_RS09115	8,553	*Glycoside hydrolase, family 4*
CTN_RS00200	8,389	*Putative uncharacterized protein*
CTN_RS01610	8,305	*Phospho-2-dehydro-3-deoxyheptonate aldolase*
CTN_RS00565	8,300	*Acetolactate synthase*
CTN_RS01075	8,136	*CTN_trnaTyr1*
CTN_RS01340	8,039	*glutamine amidotransferase, class-II*
CTN_RS03220	7,999	*FeoA family protein*
CTN_RS07130	7,879	*Oligopeptide ABC transporter, ATP-binding protein*
CTN_RS06530	7,653	*Putative uncharacterized protein*
CTN_RS01745	7,622	*Membrane protein-like protein*
CTN_RS08970	7,581	*Alkyl hydroperoxide reductase/ Thiol specific antioxidant/ Mal allergen*
CTN_RS09100	7,529	*MBL fold metallo-hydrolase*
CTN_RS06525	7,516	*Biotin synthetase*
CTN_RS01360	7,476	*Glutamate synthase (NADPH) GltB3 subunit*
CTN_RS01350	7,407	*4Fe-4S ferredoxin, iron-sulfur binding domain protein*
CTN_RS00525	7,218	*3-isopropylmalate dehydrogenase*
CTN_RS04810	7,213	*Dihydrodipicolinate reductase*
CTN_RS00950	7,169	*CTN_trnaSer1*

CTN_RS06235	7,122	*CTN_trnaGly3*
CTN_RS05515	7,108	*Rubrerythrin*
CTN_RS08960	7,107	*Glycosidase*
CTN_RS01085	7,082	*CTN_trnaMet3*
CTN_RS00330	7,078	*Amino acid ABC transporter, ATP-binding protein*
CTN_RS07625	6,990	*Histidinol-phosphate aminotransferase*
CTN_RS01735	6,829	*Cation-transporting ATPase, P-type*
CTN_RS01730	6,796	*Ubiquinone/menaquinone biosynthesis-related protein*
CTN_RS03865	6,690	*Putative uncharacterized protein*
CTN_RS08770	6,567	*Uracil-xanthine permease*
CTN_RS04815	6,538	*2,3,4,5-tetrahydropyridine-2-carboxylate N-succinyltransferase-related protein*
CTN_RS04290	6,507	*CTN_trnaAla1*
CTN_RS04300	6,499	*50S ribosomal protein L31*
CTN_RS03990	6,486	*Glutamyl-tRNA synthetase 2*
CTN_RS00535	6,423	*3-isopropylmalate dehydratase large subunit 2*
CTN_RS00530	6,400	*3-isopropylmalate dehydratase small subunit 2*
CTN_RS06535	6,395	*FeFe] hydrogenase H-cluster radical SAM maturase HydG*
CTN_RS01070	6,384	*CTN_trnaTrp1*
CTN_RS07620	6,360	*Histidinol dehydrogenase*
CTN_RS07145	6,355	*Isocitrate dehydrogenase*
CTN_RS04305	6,323	*RNA binding S1 domain protein*
CTN_RS05920	6,266	*Antibiotic ABC transporter, ATP-binding protein*
CTN_RS07800	6,257	*Putative uncharacterized protein*
CTN_RS01940	6,159	*Citrate synthase*
CTN_RS06520	6,151	*Cystathionine gamma-synthase*
CTN_RS04395	6,143	*sulfatase*
CTN_RS08145	6,102	*hypothetical protein*
CTN_RS00265	6,097	*30S ribosomal protein S18*
CTN_RS08455	6,076	*Homoserine O-succinyltransferase*
CTN_RS09105	6,005	*Oxidoreductase*
CTN_RS01345	5,985	*Glutamate synthase (NADPH) GltB2 subunit*
CTN_RS06540	5,976	*iron-only hydrogenase system regulator*
CTN_RS08225	5,961	*ferredoxin*

CTN_RS05860	5,955	*Thioredoxin*
CTN_RS06070	5,937	*CTN_trnaCys1*
CTN_RS06095	5,923	*FeS assembly protein SufD*
CTN_RS01595	5,912	*shikimate dehydrogenase*
CTN_RS01750	5,902	*YHS domain protein*
CTN_RS00550	5,832	*Dihydroxy-acid dehydratase*
CTN_RS06100	5,824	*Iron-regulated ABC transporter membrane component SufB*
CTN_RS00625	5,771	*Cation efflux system protein*
CTN_RS06090	5,738	*cysteine desulfurase*
CTN_RS04885	5,721	*30S ribosomal protein S12*
CTN_RS04910	5,691	*50S ribosomal protein L3*
CTN_RS04915	5,655	*50S ribosomal protein L4*
CTN_RS00325	5,646	*Amino acid ABC transporter, permease protein*
CTN_RS09110	5,604	*Menaquinone biosynthesis methyltransferase ubiE*
CTN_RS07530	5,564	*Major facilitator superfamily MFS_1*
CTN_RS04550	5,535	*V-ATPase D-subunit*
CTN_RS04895	5,526	*Elongation factor G*
CTN_RS00750	5,522	*Prolyl-tRNA synthetase*
CTN_RS07910	5,512	*Putative uncharacterized protein*
CTN_RS07115	5,503	*6-phosphogluconolactonase*
CTN_RS07180	5,500	*Branched chain amino acid ABC transporter, permease protein*
CTN_RS01740	5,457	*Alkylhydroperoxidase like protein, AhpD family*
CTN_RS00005	5,410	*Cupin 2, conserved barrel domain protein*
CTN_RS01040	5,406	*50S ribosomal protein L7/L12*
CTN_RS07175	5,359	*Branched chain amino acid ABC transporter, permease protein*
CTN_RS09475	5,333	*Peptidase M50*
CTN_RS01355	5,302	*NADH oxidase*
CTN_RS00540	5,278	*2-isopropylmalate synthase*
CTN_RS00555	5,268	*Ketol-acid reductoisomerase*
CTN_RS08450	5,215	*O-acetylhomoserine sulfhydrylase*
CTN_RS04825	5,184	*Diaminopimelate decarboxylase*
CTN_RS07155	5,141	*Cytochrome C-type biogenesis protein*
CTN_RS05460	5,030	*Putative uncharacterized protein*

CTN_RS04880	5,029	*Putative uncharacterized protein*
CTN_RS07025	4,980	*Transcriptional regulator, Crp/Fnr family*
CTN_RS00465	4,972	*Putative uncharacterized protein*
CTN_RS06450	4,932	*Cupin 2, conserved barrel domain protein*
CTN_RS06430	4,917	*Multidrug resistance protein*
CTN_RS01600	4,895	*3-phosphoshikimate 1-carboxyvinyltransferase*
CTN_RS07110	4,842	*Glucose-6-phosphate 1-dehydrogenase*
CTN_RS00470	4,831	*Sugar fermentation stimulation protein like protein*
CTN_RS00275	4,829	*30S ribosomal protein S6*
CTN_RS01605	4,823	*Prephenate dehydrogenase*
CTN_RS00500	4,810	*Magnesium transport protein corA*
CTN_RS03365	4,774	*Hypothetical Protein*
CTN_RS05930	4,739	*Aspartate aminotransferase*
CTN_RS05915	4,715	*Antibiotic ABC transporter, transmembrane protein*
CTN_RS00515	4,697	*Carbamoyl-phosphate synthase small chain*
CTN_RS04905	4,696	*30S ribosomal protein S10*
CTN_RS03075	4,678	*Iron(III) ABC transporter, ATP-binding protein*
CTN_RS00545	4,676	*putative alpha-isopropylmalate/homocitrate synthase family transferase*
CTN_RS03215	4,657	*Iron(II) transport protein B*
CTN_RS07900	4,646	*Redox-active disulfide protein 2*
CTN_RS00015	4,630	*Spermidine synthase*
CTN_RS05455	4,598	*CTN_trnaGlu1*
CTN_RS08600	4,517	*Translation factor SUA5*
CTN_RS00915	4,461	*CTN_trnaGly1*
CTN_RS05925	4,449	*D-isomer specific 2-hydroxyacid dehydrogenase, NAD-binding*
CTN_RS02785	4,417	*Tryptophan synthase alpha chain*
CTN_RS04920	4,410	*50S ribosomal protein L23*
CTN_RS04280	4,407	*CTN_trnaLys2*
CTN_RS04520	4,404	*Putative A-ATPase I-subunit*
CTN_RS05835	4,391	*CTN_trnaPhe1*
CTN_RS09060	4,364	*30S ribosomal protein S2*
CTN_RS07185	4,331	*Branched chain amino acid ABC transporter, periplasmic amino acid-binding protein*
CTN_RS03745	4,290	*Riboflavin biosynthesis protein RibD*

CTN_RS08760	4,256	*tRNA-Ser*
CTN_RS01335	4,234	*CTN_trnaGln1*
CTN_RS01935	4,201	*3-isopropylmalate dehydratase large subunit 1*
CTN_RS01080	4,184	*CTN_trnaThr2*
CTN_RS04790	4,177	*hypothetical protein*
CTN_RS00095	4,175	*DUF1659 domain-containing protein*
CTN_RS09560	4,163	*Serine acetyltransferase*
CTN_RS06215	4,144	*Inosine-5'-monophosphate dehydrogenase*
CTN_RS00475	4,116	*Conserved protein/domain typically associated with flavoprotein oxygenase DIM6/NTAB family-like protein*
CTN_RS09480	4,056	*Polysaccharide pyruvyl transferase*
CTN_RS04545	4,036	*V-ATPase B-subunit*
CTN_RS00755	4,035	*ComM protein*
CTN_RS03685	4,020	*Dinitrogenase iron-molybdenum cofactor biosynthesis protein*
CTN_RS04820	3,976	*Aspartokinase*
CTN_RS00920	3,922	*Putative uncharacterized protein*
CTN_RS03070	3,916	*Iron(III) ABC transporter, permease protein*
CTN_RS00495	3,902	*Methyltransferase type 11*
CTN_RS05525	3,895	*1,4-dihydroxy-2-naphthoate octaprenyltransferase*
CTN_RS08135	3,865	*Putative uncharacterized protein*
CTN_RS01585	3,823	*Bifunctional shikimate kinase/3-dehydroquinate synthase*
CTN_RS07085	3,809	*Putative uncharacterized protein*
CTN_RS04900	3,791	*Elongation factor Tu*
CTN_RS05875	3,785	*Putative uncharacterized protein*
CTN_RS04955	3,783	*30S ribosomal protein S17*
CTN_RS04285	3,774	*CTN_trnaArg5*
CTN_RS04805	3,768	*Dihydrodipicolinate synthase*
CTN_RS00010	3,763	*S-adenosylmethionine decarboxylase proenzyme*
CTN_RS08920	3,747	*Phosphomethylpyrimidine kinase type-1*
CTN_RS03705	3,726	*GMP synthase*
CTN_RS05990	3,719	*CTN_trnaVal3*
CTN_RS04945	3,714	*50S ribosomal protein L16*
CTN_RS09065	3,711	*Major facilitator superfamily MFS_1*
CTN_RS01530	3,709	*Nitroreductase*

CTN_RS06065	3,696	*Spermidine/putrescine ABC transporter, periplasmic spermidine/putrescine-binding protein*
CTN_RS08605	3,695	*CTN_trnaLeu4*
CTN_RS05900	3,683	*Argininosuccinate lyase*
CTN_RS08965	3,665	*Putative uncharacterized protein*
CTN_RS00520	3,651	*Carbamoyl-phosphate synthase large chain*
CTN_RS06105	3,618	*FeS assembly ATPase SufC*
CTN_RS00570	3,616	*Aspartokinase II*
CTN_RS07890	3,614	*Heavy metal resistance transcription regulator*
CTN_RS01485	3,606	*Arginine repressor*
CTN_RS07350	3,568	*dTMP kinase*
CTN_RS07135	3,540	*Oligopeptide ABC transporter, periplasmic oligopeptide-binding protein*
CTN_RS00250	3,527	*Putative uncharacterized protein*
CTN_RS02780	3,518	*Tryptophan synthase beta chain 1*
CTN_RS01590	3,514	*Chorismate synthase*
CTN_RS05905	3,502	*Argininosuccinate synthase*
CTN_RS05055	3,500	*50S ribosomal protein L17*
CTN_RS05150	3,479	*50S ribosomal protein L13*
CTN_RS04000	3,479	*Radical SAM domain protein*
CTN_RS08560	3,463	*Protein-export membrane protein SecD precursor*
CTN_RS03740	3,460	*Riboflavin synthase, alpha subunit*
CTN_RS00580	3,432	*Homoserine kinase*
CTN_RS04985	3,419	*50S ribosomal protein L6*
CTN_RS04960	3,418	*50S ribosomal protein L14*
CTN_RS04780	3,413	*Hypothetical Protein*
CTN_RS08730	3,407	*ABC transporter ATP-binding protein*
CTN_RS07080	3,401	*Esterase*
CTN_RS04965	3,394	*50S ribosomal protein L24*
CTN_RS00270	3,389	*Single-stranded DNA-binding protein*
CTN_RS05895	3,367	*N-acetyl-gamma-glutamyl-phosphate reductase*
CTN_RS08925	3,365	*Xylose isomerase domain protein TIM barrel*
CTN_RS08570	3,364	*Putative uncharacterized protein precursor*
CTN_RS00585	3,349	*ABC transporter, ATP-binding protein*
CTN_RS04530	3,343	*V-ATPase G-subunit*

CTN_RS09595	3,331	*Rubredoxin-type Fe(Cys)4 protein*
CTN_RS04540	3,295	*V-ATPase A-subunit*
CTN_RS05565	3,282	*Putative uncharacterized protein*
CTN_RS00895	3,256	*ABC transporter related*
CTN_RS01640	3,254	*dihydroorotase*
CTN_RS00480	3,253	*Major facilitator superfamily MFS_1*
CTN_RS09585	3,237	*Putative uncharacterized protein*
CTN_RS08555	3,225	*Protein-export membrane protein SecF*
CTN_RS04950	3,218	*50S ribosomal protein L29*
CTN_RS04260	3,217	*Cobyrinic acid a,c-diamide synthase*
CTN_RS06055	3,217	*Spermidine/putrescine ABC transporter, permease protein*
CTN_RS08845	3,216	*Lipophilic protein*
CTN_RS09565	3,209	*Cysteine synthase*
CTN_RS04785	3,205	*CTN_trnaSer3*
CTN_RS05265	3,196	*Glycerol uptake operon antiterminator*
CTN_RS06220	3,170	*Peptidase M16 domain protein*
CTN_RS04980	3,164	*30S ribosomal protein S8*
CTN_RS03600	3,160	*S-adenosylmethionine synthetase*
CTN_RS05890	3,152	*Arginine biosynthesis bifunctional protein argJ*
CTN_RS01100	3,152	*Putative uncharacterized protein*
CTN_RS08930	3,145	*Thiamine biosynthesis protein thiC*
CTN_RS04200	3,139	*Elongation factor Ts*
CTN_RS09590	3,106	*Putative uncharacterized protein*
CTN_RS06060	3,104	*Spermidine/putrescine import ATP-binding protein potA*
CTN_RS06925	3,096	*Galactokinase*
CTN_RS08515	3,093	*Thioredoxin reductase*
CTN_RS05830	3,093	*CTN_trnaVal2*
CTN_RS08750	3,073	*Phenylalanyl-tRNA synthetase beta chain*
CTN_RS03385	3,070	*Pyruvate synthase subunit porA*
CTN_RS03370	3,048	*Protein crcB like protein*
CTN_RS06050	3,044	*Spermidine/putrescine ABC transporter, permease protein*
CTN_RS00370	3,042	*Rhomboid family protein precursor*
CTN_RS02435	3,042	*Putative uncharacterized protein*

CTN_RS04400	3,038	*Putative uncharacterized protein*
CTN_RS05000	3,026	*Ribosomal protein L30*
CTN_RS09300	3,024	*Putative uncharacterized protein*
CTN_RS04925	3,010	*50S ribosomal protein L2*
CTN_RS08390	3,008	*Beta-lactamase domain protein*
CTN_RS00320	2,997	*Amino acid ABC transporter, periplasmic amino acid-binding protein*
CTN_RS05155	2,994	*30S ribosomal protein S9*
CTN_RS03390	2,990	*Pyruvate synthase subunit porD*
CTN_RS03805	2,977	*Beta transducin-related protein*
CTN_RS05010	2,968	*Preprotein translocase SecY subunit*
CTN_RS05145	2,968	*Membrane protein-like protein*
CTN_RS06920	2,962	*Galactose-1-phosphate uridylyltransferase*
CTN_RS08840	2,951	*O-antigen polymerase*
CTN_RS07300	2,950	*Subunit of the Multisubunit Na+/H+ antiporter-like protein*
CTN_RS01930	2,943	*3-isopropylmalate dehydratase small subunit 1*
CTN_RS00365	2,924	*Lipopolysaccharide biosynthesis protein BplA*
CTN_RS09470	2,919	*Tartrate dehydratase beta subunit/Fumarate hydratase class I C-terminal domain-like protein*
CTN_RS04515	2,918	*H+-transporting two-sector ATPase C (AC39) subunit*
CTN_RS04890	2,910	*30S ribosomal protein S7*
CTN_RS07170	2,909	*Branched chain amino acid ABC transporter, ATP-binding protein*
CTN_RS08005	2,901	*Sensory box histidine kinase*
CTN_RS05745	2,898	*Signal peptidase I*
CTN_RS05385	2,888	*hypothetical protein*
CTN_RS03290	2,888	*Phospholipase/Carboxylesterase precursor*
CTN_RS08565	2,881	*preprotein translocase, YajC subunit*
CTN_RS00575	2,872	*Threonine synthase*
CTN_RS08935	2,867	*Putative thiazole biosynthetic enzyme*
CTN_RS00335	2,865	*Penicillin-binding protein 2*
CTN_RS05310	2,861	*Hypothetical Protein*
CTN_RS07895	2,858	*Permease*
CTN_RS06690	2,855	*Extracellular solute-binding protein, family 1 precursor*
CTN_RS06225	2,847	*Polyribonucleotide nucleotidyltransferase*
CTN_RS05140	2,840	*50S ribosomal protein L27*

CTN_RS02745	2,835	CTN_trnaPro3
CTN_RS05005	2,834	50S ribosomal protein L15
CTN_RS01725	2,813	Putative uncharacterized protein
CTN_RS08015	2,810	GCN5-related N-acetyltransferase
CTN_RS04830	2,806	Hydrolase, ama/hipO/hyuC family
CTN_RS09275	2,771	Uracil phosphoribosyltransferase
CTN_RS05275	2,767	Methyl-accepting chemotaxis protein 4
CTN_RS07105	2,757	Phage shock protein C, PspC
CTN_RS04775	2,755	N utilization substance protein A
CTN_RS08375	2,753	Stage V sporulation protein S
CTN_RS02115	2,751	50S ribosomal protein L28
CTN_RS01920	2,748	Glutamate 5-kinase
CTN_RS03950	2,744	L-lactate dehydrogenase
CTN_RS00315	2,739	Xanthine/uracil/vitamin C permease
CTN_RS09600	2,736	Putative superoxide reductase
CTN_RS02105	2,731	Metal dependent phosphohydrolase
CTN_RS05050	2,723	DNA-directed RNA polymerase subunit alpha
CTN_RS01645	2,709	dihydroorotate dehydrogenase
CTN_RS00590	2,700	ABC-2 type transporter
CTN_RS01110	2,697	Phosphoribosylaminoimidazole carboxylase catalytic subunit
CTN_RS08500	2,671	S-adenosyl-L-methionine-dependent methyltransferase mraW
CTN_RS02165	2,668	Electron transport complex, RnfABCDGE type, D subunit precursor
CTN_RS02420	2,665	CTN_trnaArg6
CTN_RS08520	2,660	Glutaredoxin-related protein
CTN_RS07305	2,658	Putative uncharacterized protein
CTN_RS05255	2,641	Oxidoreductase
CTN_RS07765	2,639	Methyl-accepting chemotaxis sensory transducer precursor
CTN_RS01045	2,635	50S ribosomal protein L10
CTN_RS08370	2,633	Putative uncharacterized protein
CTN_RS02765	2,629	Anthranilate synthase component II
CTN_RS04265	2,620	50S ribosomal protein L20
CTN_RS01095	2,608	Thymidylate synthase thyX
CTN_RS03395	2,608	Pyruvate synthase subunit porC

CTN_RS05560	2,605	*Polyprenyl synthetase*
CTN_RS08175	2,599	*Transcriptional regulator, PadR-like family*
CTN_RS01520	2,597	*ABC-2 type transporter*
CTN_RS08180	2,597	*Amidohydrolase*
CTN_RS07395	2,565	*Potassium uptake protein, TrkH family*
CTN_RS06255	2,558	*Rieske (2Fe-2S) domain protein*
CTN_RS01720	2,548	*Putative uncharacterized protein*
CTN_RS03680	2,547	*ferredoxin family protein*
CTN_RS02735	2,544	*O-sialoglycoprotein endopeptidase*
CTN_RS05335	2,538	*SufBD protein*
CTN_RS03720	2,534	*HflC protein precursor*
CTN_RS05120	2,519	*Jag protein*
CTN_RS02160	2,518	*Electron transport complex, RnfABCDGE type, G subunit precursor*
CTN_RS05740	2,510	*50S ribosomal protein L19*
CTN_RS05060	2,505	*Transcription termination factor rho*
CTN_RS07195	2,503	*Putative uncharacterized protein*
CTN_RS09280	2,502	*Serine hydroxymethyltransferase*
CTN_RS04270	2,496	*50S ribosomal protein L35*
CTN_RS01420	2,491	*Glycosyl transferase, group 1*
CTN_RS04970	2,489	*50S ribosomal protein L5*
CTN_RS02110	2,489	*Putative uncharacterized protein*
CTN_RS07295	2,482	*Na+/H+ antiporter MnhB subunit-related protein*
CTN_RS05710	2,474	*Signal recognition particle protein*
CTN_RS04210	2,469	*Major facilitator superfamily MFS_1*
CTN_RS06230	2,460	*30S ribosomal protein S15*
CTN_RS03210	2,453	*Putative uncharacterized protein*
CTN_RS04845	2,453	*Hypothetical Protein*
CTN_RS08395	2,450	*Undecaprenyl-diphosphatase*
CTN_RS03725	2,447	*Putative uncharacterized protein*
CTN_RS05305	2,440	*4Fe-4S ferredoxin, iron-sulfur binding domain protein*
CTN_RS04275	2,428	*Translation initiation factor IF-3*
CTN_RS09605	2,427	*Rubrerythrin*
CTN_RS03375	2,419	*oxidoreductase*

CTN_RS09260	2,412	*Na+/Ca+ antiporter, CaCA family precursor*
CTN_RS04840	2,410	*Putative uncharacterized protein*
CTN_RS04850	2,406	*Sun protein*
CTN_RS03545	2,398	*Diguanylate cyclase*
CTN_RS09465	2,383	*Lytic transglycosylase, catalytic precursor*
CTN_RS06680	2,380	*ATP-dependent DNA helicase*
CTN_RS07100	2,379	*Putative uncharacterized protein*
CTN_RS01370	2,360	*Glycosyl transferase, group 1*
CTN_RS06665	2,354	*Putative uncharacterized protein*
CTN_RS04940	2,348	*30S ribosomal protein S3*
CTN_RS03695	2,336	*Chromate transport protein*
CTN_RS07280	2,334	*Putative uncharacterized protein precursor*
CTN_RS05950	2,331	*Alanyl-tRNA synthetase*
CTN_RS05185	2,324	*Hypothetical Protein*
CTN_RS02775	2,322	*N-(5'-phosphoribosyl)anthranilate isomerase*
CTN_RS07150	2,317	*Thioredoxin-related protein-like protein precursor*
CTN_RS07190	2,312	*Putative uncharacterized protein*
CTN_RS05880	2,308	*aspartate aminotransferase family protein*
CTN_RS02770	2,306	*Indole-3-glycerol phosphate synthase*
CTN_RS06250	2,304	*Putative uncharacterized protein precursor*
CTN_RS01105	2,303	*Phosphoribosylaminoimidazole carboxylase*
CTN_RS00645	2,300	*RNA polymerase, sigma-24 subunit, ECF subfamily*
CTN_RS01925	2,289	*Gamma-glutamyl phosphate reductase*
CTN_RS08190	2,287	*Citrate transporter*
CTN_RS05135	2,274	*Putative uncharacterized protein*
CTN_RS08445	2,271	*Type III pantothenate kinase*
CTN_RS03065	2,255	*Iron(III) ABC transporter, periplasmic-binding protein*
CTN_RS06245	2,253	*DNA-(Apurinic or apyrimidinic site) lyase / Pyrimidine dimer DNA glycosylase*
CTN_RS07540	2,231	*Ferredoxin-dependent glutamate synthase*
CTN_RS06930	2,231	*Diguanylate cyclase*
CTN_RS07340	2,223	*Phosphodiesterase, MJ0936 family*
CTN_RS06425	2,221	*Ribonuclease H*
CTN_RS07200	2,218	*MoxR protein*

CTN_RS08895	2,208	*Sugar kinase, pfkB family*
CTN_RS08715	2,201	*MiaB-like tRNA modifying enzyme*
CTN_RS03850	2,197	*Beta-glucosidase A*
CTN_RS06775	2,191	*Glutamate synthase, beta subunit*
CTN_RS05820	2,190	*hypothetical protein*
CTN_RS08755	2,190	*locus_tag=CTN_RS08755*
CTN_RS05345	2,187	*Inositol-1-monophosphatase*
CTN_RS06635	2,184	*Amidophosphoribosyltransferase*
CTN_RS05110	2,184	*Hypothetical Protein*
CTN_RS05065	2,181	*Glucokinase*
CTN_RS02440	2,164	*CTN_trnaGly2*
CTN_RS04560	2,160	*Putative uncharacterized protein*
CTN_RS05610	2,158	*CTN_trnaArg4*
CTN_RS00655	2,156	*Binding-protein-dependent transport systems inner membrane component*
CTN_RS09430	2,155	*Methyltransferase type 11*
CTN_RS07095	2,152	*Glutamine amidotransferase class-I*
CTN_RS09270	2,142	*Ribonuclease P*
CTN_RS05695	2,132	*Putative uncharacterized protein precursor*
CTN_RS03735	2,127	*3,4-dihydroxy-2-butanone-4-phosphate synthase/GTP cyclohydrolase II*
CTN_RS08955	2,126	*Putative uncharacterized protein*
CTN_RS04205	2,123	*Uridylate kinase*
CTN_RS09285	2,121	*Cysteinyl-tRNA synthetase*
CTN_RS07345	2,116	*ABC-2 type transporter precursor*
CTN_RS04975	2,115	*30S ribosomal protein S14 type Z*
CTN_RS05015	2,113	*Adenylate kinase*
CTN_RS08980	2,107	*Putative uncharacterized protein precursor*
CTN_RS03710	2,106	*N-glycosylase/DNA lyase*
CTN_RS04525	2,092	*V-ATPase F-subunit*
CTN_RS09570	2,088	*Putative uncharacterized protein*
CTN_RS00280	2,087	*Iron-dependent transcriptional repressor*
CTN_RS05350	2,084	*Beta-fructosidase*
CTN_RS02095	2,084	*D-alanine--D-alanine ligase*
CTN_RS05945	2,080	*Phosphatidate cytidylyltransferase*

CTN_RS00985	2,068	*Uncharacterized protein*
CTN_RS01035	2,068	*DNA-directed RNA polymerase subunit beta*
CTN_RS08380	2,065	*Galactose-1-phosphate uridylyltransferase*
CTN_RS00360	2,060	*Putative uncharacterized protein*
CTN_RS01470	2,057	*MFS transporter*
CTN_RS02880	2,056	*Radical SAM domain protein*
CTN_RS09295	2,055	*Biotin/lipoyl attachment domain-containing protein*
CTN_RS06495	2,046	*Putative uncharacterized protein*
CTN_RS09305	2,045	*Propionyl-CoA carboxylase, beta subunit*
CTN_RS08220	2,044	*Uncharacterized protein*
CTN_RS06005	2,043	*Dephospho-CoA kinase*
CTN_RS07020	2,042	*Hydroxylamine reductase*
CTN_RS05125	2,029	*Cupin 2, conserved barrel domain protein*
CTN_RS04835	2,026	*Ferric uptake regulation protein*
CTN_RS00410	2,015	*GCN5-related N-acetyltransferase*
CTN_RS04555	2,014	*Alanyl-tRNA synthetase-related protein*
CTN_RS03955	2,011	*Cobalt transport protein*
CTN_RS02475	2,009	*CTN_trnaAla3*
CTN_RS07310	2,009	*Putative uncharacterized protein*
CTN_RS07245	2,001	*Flagellar hook-associated protein 2*
CTN_RS05100	1,993	*50S ribosomal protein L34*
CTN_RS03605	1,988	*30S ribosomal protein S20*
CTN_RS02675	1,986	*Putative uncharacterized protein precursor*
CTN_RS08150	1,984	*Putative uncharacterized protein precursor*
CTN_RS07980	1,980	*SIrA family protein*
CTN_RS01005	1,972	*Putative uncharacterized protein*
CTN_RS07275	1,966	*Putative uncharacterized protein precursor*
CTN_RS07845	1,965	*Major facilitator superfamily MFS_1 precursor*
CTN_RS07010	1,962	*Ferredoxin*
CTN_RS05720	1,962	*Hypothetical Protein*
CTN_RS07390	1,951	*Histidyl-tRNA synthetase*
CTN_RS00675	1,947	*Methionyl-tRNA formyltransferase*
CTN_RS06500	1,946	*Aspartyl/glutamyl-tRNA(Asn/Gln) amidotransferase subunit B*

CTN_RS02760	1,945	*Anthranilate synthase component 1*
CTN_RS07400	1,941	*TrkA-N domain protein*
CTN_RS00405	1,938	*Pyrroline-5-carboxylate reductase*
CTN_RS02155	1,927	*Electron transport complex, RnfABCDGE type, E subunit*
CTN_RS00445	1,918	*Signal recognition particle-docking protein FtsY*
CTN_RS05070	1,912	*DegV family protein*
CTN_RS08805	1,909	*Glutamine--fructose-6-phosphate transaminase*
CTN_RS09120	1,908	*CTN_trnaGln2*
CTN_RS05045	1,892	*30S ribosomal protein S4*
CTN_RS04535	1,889	*V-ATPase E-subunit*
CTN_RS01650	1,889	*Dihydroorotate dehydrogenase*
CTN_RS05180	1,888	*hypothetical protein*
CTN_RS06075	1,881	*Phosphoglycerate mutase*
CTN_RS09620	1,874	*Putative uncharacterized protein precursor*
CTN_RS06355	1,873	*Tetratricopeptide TPR_2 repeat protein*
CTN_RS00255	1,872	*Beta-lactamase domain protein*
CTN_RS04415	1,868	*Transcriptional repressor nrdR*
CTN_RS00885	1,867	*ABC transporter, permease protein, cysTW family*
CTN_RS07605	1,863	*Citrate transporter*
CTN_RS00995	1,859	*Regulatory protein*
CTN_RS02235	1,858	*UDP-N-acetylmuramate--L-alanine ligase*
CTN_RS04495	1,854	*Amidohydrolase*
CTN_RS07410	1,850	*Rhomboid family protein*
CTN_RS07355	1,850	*Ornithine carbamoyltransferase*
CTN_RS08940	1,849	*Putative uncharacterized protein*
CTN_RS04935	1,847	*50S ribosomal protein L22*
CTN_RS01845	1,840	*Glucose-6-phosphate isomerase*
CTN_RS02740	1,840	*Regulatory protein, FmdB family*
CTN_RS05980	1,838	*Putative uncharacterized protein*
CTN_RS05280	1,833	*Redox-sensing transcriptional repressor rex 2*
CTN_RS00650	1,824	*Binding-protein-dependent transport systems inner membrane component*
CTN_RS06670	1,823	*GTP-binding protein YchF*
CTN_RS02100	1,822	*DNA topoisomerase 1*

CTN_RS08170	1,820	*Methyltransferase type 11*
CTN_RS07205	1,819	*Aminotransferase*
CTN_RS03450	1,813	*DNA helicase*
CTN_RS04700	1,810	*Transketolase*
CTN_RS05115	1,809	*Inner membrane protein oxaA*
CTN_RS02480	1,807	*Putative iron(III) ABC transporter, ATP-binding protein*
CTN_RS01575	1,795	*Lysine exporter protein (LYSE/YGGA) precursor*
CTN_RS01050	1,793	*50S ribosomal protein L1*
CTN_RS04185	1,792	*DegV family protein*
CTN_RS06915	1,786	*Alpha-galactosidase*
CTN_RS01525	1,784	*ABC-2 type transporter*
CTN_RS07210	1,784	*Phosphate acetyltransferase*
CTN_RS05285	1,777	*Fe-hydrogenase alpha subunit*
CTN_RS02085	1,776	*Phosphate permease*
CTN_RS03715	1,775	*HflK protein precursor*
CTN_RS04020	1,771	*Glutamate synthase, beta subunit*
CTN_RS02620	1,768	*Leucyl-tRNA synthetase*
CTN_RS07075	1,765	*Response regulator receiver protein*
CTN_RS06415	1,762	*Putative uncharacterized protein*
CTN_RS05550	1,758	*4Fe-4S ferredoxin iron-sulfur binding domain protein*
CTN_RS06280	1,756	*ABC transporter ATP-binding protein*
CTN_RS08385	1,754	*glycogen synthase*
CTN_RS04015	1,753	*Dihydrofolate reductase*
CTN_RS06335	1,750	*hypothetical protein*
CTN_RS07650	1,749	*Histidine biosynthesis bifunctional protein hisIE*
CTN_RS02040	1,749	*Methylenetetrahydrofolate reductase*
CTN_RS04360	1,746	*Type IV prepilin peptidase*
CTN_RS00890	1,743	*Pyrimidine biosynthesis enzyme*
CTN_RS03730	1,743	*6,7-dimethyl-8-ribityllumazine synthase*
CTN_RS02690	1,742	*Prephenate dehydratase*
CTN_RS02515	1,736	*Phosphoglucosamine mutase*
CTN_RS08830	1,734	*Transcriptional regulator XylF*
CTN_RS00980	1,734	*Integral membrane protein-like protein*

CTN_RS04505	1,730	*Metallophosphoesterase*
CTN_RS09265	1,724	*Putative uncharacterized protein*
CTN_RS02670	1,721	*Prolipoprotein diacylglyceryl transferase*
CTN_RS06000	1,708	*Methyltransferase*
CTN_RS05030	1,707	*Hypothetical Protein*
CTN_RS06910	1,706	*beta-galactosidase*
CTN_RS03590	1,698	*Peptidase M23B precursor*
CTN_RS00825	1,698	*Putative uncharacterized protein*
CTN_RS03380	1,696	*Pyruvate synthase subunit porB*
CTN_RS00835	1,695	*PhoH-related protein*
CTN_RS04770	1,690	*Ferric uptake regulation protein*
CTN_RS05545	1,687	*FAD dependent oxidoreductase precursor*
CTN_RS06285	1,687	*ABC transporter, ATP-binding protein*
CTN_RS05380	1,685	*Putative uncharacterized protein precursor*
CTN_RS08525	1,682	*Putative uncharacterized protein*
CTN_RS00285	1,679	*Putative uncharacterized protein*
CTN_RS02615	1,678	*Putative uncharacterized protein*
CTN_RS05635	1,678	*Putative uncharacterized protein*
CTN_RS03700	1,676	*Chromate transport protein*
CTN_RS00340	1,676	*Putative uncharacterized protein precursor*
CTN_RS00065	1,675	*Putative uncharacterized protein*
CTN_RS07525	1,664	*Putative uncharacterized protein*
CTN_RS06330	1,662	*Hypothetical Protein*
CTN_RS02490	1,657	*Putative iron(III) ABC transporter, periplasmic iron-binding protein*
CTN_RS05035	1,655	*30S ribosomal protein S13*
CTN_RS01055	1,652	*50S ribosomal protein L11*
CTN_RS01540	1,652	*Anaerobic ribonucleoside-triphosphate reductase activating protein*
CTN_RS04190	1,642	*Sigma 54 modulation protein / SSU ribosomal protein S30P*
CTN_RS08575	1,641	*Riboflavin kinase/FMN adenylyltransferase*
CTN_RS04470	1,639	*Ribulose-phosphate 3-epimerase*
CTN_RS06370	1,637	*hypothetical protein*
CTN_RS01535	1,636	*Anaerobic ribonucleoside-triphosphate reductase class III*
CTN_RS01950	1,634	*ABC transporter, transmembrane region*

CTN_RS06640	1,634	*Phosphoribosylformylglycinamidine synthase II*
CTN_RS07235	1,632	*Putative uncharacterized protein*
CTN_RS08790	1,631	*Transcriptional regulator, MarR family*
CTN_RS08800	1,628	*N-acetylglucosamine-6-phosphate deacetylase*
CTN_RS00880	1,627	*Putative uncharacterized protein*
CTN_RS08855	1,626	*histidinol-phosphatase*
CTN_RS00025	1,622	*Putative uncharacterized protein*
CTN_RS05270	1,621	*Glycerol kinase 2*
CTN_RS05700	1,620	*MscS Mechanosensitive ion channel*
CTN_RS01510	1,618	*Putative uncharacterized protein precursor*
CTN_RS05885	1,612	*Acetylglutamate kinase*
CTN_RS02855	1,611	*Ferric uptake regulation protein*
CTN_RS00635	1,611	*Putative uncharacterized protein*
CTN_RS03935	1,609	*Uncharacterized protein*
CTN_RS00975	1,609	*putative diguanylate cyclase*
CTN_RS00400	1,607	*CTN_trnaThr3*
CTN_RS01030	1,602	*DNA-directed RNA polymerase subunit beta'*
CTN_RS04860	1,600	*Uncharacterized conserved protein*
CTN_RS01840	1,598	*Betaine-aldehyde dehydrogenase*
CTN_RS01450	1,587	*4-alpha-glucanotransferase*
CTN_RS08440	1,582	*Methyltransferase*
CTN_RS02595	1,582	*Reverse gyrase*
CTN_RS04405	1,582	*Lysyl-tRNA synthetase*
CTN_RS05040	1,579	*30S ribosomal protein S11*
CTN_RS07670	1,577	*Glutaredoxin-like protein, YruB-family*
CTN_RS03995	1,576	*Phosphodiesterase, MJ0936 family*
CTN_RS07320	1,573	*NADH/Ubiquinone/plastoquinone (Complex I) precursor*
CTN_RS04990	1,570	*50S ribosomal protein L18*
CTN_RS04410	1,560	*Transcription elongation factor greA*
CTN_RS00375	1,559	*Lipopolysaccharide biosynthesis protein*
CTN_RS00810	1,556	*Oligopeptide ABC transporter, ATP-binding protein*
CTN_RS05330	1,551	*ABC transporter, ATP-binding protein*
CTN_RS00680	1,548	*GTP-binding protein HflX*

CTN_RS08700	1,547	*DNA gyrase subunit B*
CTN_RS09010	1,546	*Radical SAM domain protein*
CTN_RS00020	1,545	*tRNA-i(6)A37 thiotransferase enzyme*
CTN_RS02940	1,545	*Sugar ABC transporter, permease protein*
CTN_RS05730	1,542	*tRNA (guanine-N(1)-)-methyltransferase*
CTN_RS04195	1,540	*Ribonuclease, Rne/Rng family*
CTN_RS07405	1,537	*TrkA-N domain protein precursor*
CTN_RS08695	1,536	*Putative uncharacterized protein*
CTN_RS01825	1,536	*Alcohol dehydrogenase GroES domain protein*
CTN_RS07415	1,533	*Putative uncharacterized protein*
CTN_RS03580	1,533	*5'-nucleotidase surE*
CTN_RS04510	1,532	*Putative uncharacterized protein*
CTN_RS02865	1,529	*Oxidoreductase*
CTN_RS04995	1,528	*30S ribosomal protein S5*
CTN_RS06695	1,523	*Binding-protein-dependent transport systems inner membrane component precursor*
CTN_RS02485	1,516	*Putative, iron(III) ABC transporter, permease protein*
CTN_RS08885	1,510	*2-phosphosulfolactate phosphatase*
CTN_RS04215	1,508	*3H domain protein*
CTN_RS08215	1,506	*Uncharacterized protein*
CTN_RS04025	1,502	*dihydroorotate dehydrogenase electron transfer subunit*
CTN_RS05170	1,500	*transcription-repair coupling factor*
CTN_RS07645	1,499	*Imidazole glycerol phosphate synthase subunit hisF*
CTN_RS08510	1,498	*Penicillin-binding protein 2*
CTN_RS01010	1,497	*Chemotaxis protein methyltransferase*
CTN_RS03205	1,494	*Esterase*
CTN_RS04930	1,492	*30S ribosomal protein S19*
CTN_RS07455	1,490	*Putative uncharacterized protein*
CTN_RS03125	1,490	*Mannonate dehydratase*
CTN_RS05955	1,489	*Putative uncharacterized protein*
CTN_RS07515	1,489	*Putative uncharacterized protein*
CTN_RS06085	1,487	*NifU-like protein*
CTN_RS03970	1,485	*Putative uncharacterized protein*
CTN_RS06655	1,485	*phosphoribosylaminoimidazolesuccinocarboxamide synthase*

CTN_RS01060	1,483	*Transcription antitermination protein nusG*
CTN_RS03960	1,482	*Peptidase S16, lon domain protein*
CTN_RS05130	1,480	*50S ribosomal protein L21*
CTN_RS08795	1,479	*multidrug resistance protein norM*
CTN_RS08125	1,476	*Putative uncharacterized protein*
CTN_RS07315	1,476	*Putative uncharacterized protein*
CTN_RS05025	1,473	*Translation initiation factor IF-1*
CTN_RS07635	1,470	*Imidazole glycerol phosphate synthase subunit hisH*
CTN_RS03965	1,467	*Septum site-determining protein minD*
CTN_RS06945	1,459	*Putative uncharacterized protein*
CTN_RS09435	1,451	*GatB/Yqey domain protein*
CTN_RS05725	1,449	*16S rRNA-processing protein rimM*
CTN_RS04645	1,448	*L-allo-threonine aldolase*
CTN_RS02415	1,447	*Putative guanosine pentaphosphate phosphohydrolase*
CTN_RS02495	1,444	*Putative uncharacterized protein*
CTN_RS05195	1,443	*30S ribosomal protein S1*
CTN_RS06360	1,443	*type II toxin-antitoxin system HicB family*
CTN_RS09540	1,442	*Putative uncharacterized protein*
CTN_RS00830	1,442	*DNA polymerase III, epsilon subunit*
CTN_RS02430	1,442	*ABC transporter, ATP-binding protein*
CTN_RS02045	1,441	*methionine synthase*
CTN_RS00430	1,441	*Apolipoprotein N-acyltransferase*
CTN_RS06675	1,429	*Acid phosphatase/vanadium-dependent haloperoxidase related*
CTN_RS05715	1,425	*30S ribosomal protein S16*
CTN_RS01325	1,424	*Sensor protein*
CTN_RS05825	1,420	*Diguanylate cyclase*
CTN_RS01830	1,418	*Putative uncharacterized protein*
CTN_RS03235	1,417	*Putative uncharacterized protein precursor*
CTN_RS01625	1,416	*Uncharacterized protein*
CTN_RS05160	1,414	*DNA primase*
CTN_RS08710	1,414	*branched-chain-amino-acid aminotransferase*
CTN_RS07640	1,406	*phosphoribosylformimino-5-aminoimidazole carboxamide ribotide isomerase*
CTN_RS04460	1,403	*PASTA domain containing protein precursor*

CTN_RS01835	1,403	*Transcriptional regulator, GntR family*
CTN_RS09575	1,402	*Hydrolase of the metallo-beta-lactamase superfamily-like protein*
CTN_RS01675	1,395	*M4C-methyltransferase*
CTN_RS05190	1,395	*ribosome biogenesis GTPase Der*
CTN_RS05390	1,389	*Putative uncharacterized protein*
CTN_RS08945	1,388	*Linocin_M18 bacteriocin protein*
CTN_RS02150	1,387	*Electron transport complex, RnfABCDGE type, A subunit*
CTN_RS01365	1,386	*Transcriptional regulator, XylR-related*
CTN_RS07360	1,385	*Adenylosuccinate synthetase*
CTN_RS06585	1,384	*ATPase-like protein*
CTN_RS04365	1,382	*SAM-dependent methyltransferase*
CTN_RS01850	1,382	*K+ channel, beta subunit*
CTN_RS05105	1,380	*ribonuclease P protein component*
CTN_RS07050	1,380	*Oxygen-independent coproporphyrinogen III oxidase*
CTN_RS03425	1,378	*Rubrerythrin*
CTN_RS08400	1,378	*CBS domain containing protein*
CTN_RS08185	1,376	*Putative signal-transduction protein with CBS domains*
CTN_RS01015	1,375	*Pseudouridine synthase*
CTN_RS02260	1,374	*Putative uncharacterized protein*
CTN_RS07365	1,373	*Adenylosuccinate lyase*
CTN_RS03585	1,368	*Peptide deformylase*
CTN_RS09425	1,368	*Holo-acyl-carrier-protein synthase*
CTN_RS02935	1,368	*Nuclease (RecB family)-like protein*
CTN_RS06590	1,367	*ABC transporter, ATP-binding protein*
CTN_RS02015	1,364	*Regulatory protein GntR HTH*
CTN_RS00865	1,363	*Putative uncharacterized protein*
CTN_RS03910	1,357	*Protein recA*
CTN_RS09015	1,357	*Phosphomannomutase*
CTN_RS04620	1,356	*Putative uncharacterized protein*
CTN_RS05355	1,353	*Putative uncharacterized protein*
CTN_RS05245	1,350	*CoA-binding domain protein*
CTN_RS04765	1,349	*ComEC/Rec2-related protein*
CTN_RS09125	1,348	*AAA family ATPase*

CTN_RS07520	1,348	*Beta-glucuronidase*
CTN_RS05225	1,347	*Uncharacterized protein*
CTN_RS00345	1,346	*Putative uncharacterized protein*
CTN_RS00780	1,344	*recombination factor protein RarA/unknown domain fusion protein*
CTN_RS03280	1,344	*Putative uncharacterized protein precursor*
CTN_RS01665	1,343	*Amidohydrolase 3*
CTN_RS00450	1,340	*Uncharacterized protein*
CTN_RS03245	1,340	*Aminopeptidase P*
CTN_RS05940	1,340	*Undecaprenyl pyrophosphate synthetase*
CTN_RS00815	1,339	*Putative uncharacterized protein*
CTN_RS02650	1,338	*Putative uncharacterized protein*
CTN_RS00075	1,335	*Putative uncharacterized protein precursor*
CTN_RS00245	1,334	*Putative uncharacterized protein*
CTN_RS00925	1,331	*UvrABC system protein A*
CTN_RS08670	1,329	*Rod shape-determining protein RodA*
CTN_RS00805	1,328	*Oligopeptide/dipeptide ABC transporter, ATPase subunit*
CTN_RS07450	1,327	*Ribosomal protein L11 methyltransferase*
CTN_RS09460	1,326	*DNA polymerase III, gamma and tau subunit*
CTN_RS03905	1,326	*Regulatory protein recX*
CTN_RS00385	1,320	*Thioesterase superfamily*
CTN_RS02090	1,320	*Putative uncharacterized protein*
CTN_RS02050	1,318	*5-methyltetrahydrofolate S-homocysteine methyltransferase*
CTN_RS02520	1,318	*Putative uncharacterized protein*
CTN_RS04650	1,317	*Putative uncharacterized protein*
CTN_RS02825	1,315	*Oxaloacetate decarboxylase, alpha subunit*
CTN_RS07745	1,311	*Transcriptional regulator*
CTN_RS09410	1,306	*ATP-dependent Clp protease proteolytic subunit*
CTN_RS06435	1,303	*Beta-lactamase domain protein*
CTN_RS02665	1,295	*Nucleoside-triphosphatase*
CTN_RS03155	1,290	*Putative uncharacterized protein*
CTN_RS09320	1,288	*Putative uncharacterized protein*
CTN_RS03150	1,285	*Uronate isomerase*
CTN_RS01115	1,284	*Small GTP-binding protein*

CTN_RS00855	1,282	*Putative uncharacterized protein*
CTN_RS06350	1,280	*Radical SAM domain protein*
CTN_RS05600	1,274	*Flagellar basal-body rod protein FlgF*
CTN_RS09290	1,273	*Purine-binding chemotaxis protein*
CTN_RS01120	1,271	*Fumarate lyase*
CTN_RS01875	1,271	*Alpha-xylosidase*
CTN_RS02640	1,270	*Putative uncharacterized protein*
CTN_RS05855	1,267	*Putative uncharacterized protein*
CTN_RS08405	1,267	*4-hydroxy-3-methylbut-2-en-1-yl diphosphate synthase*
CTN_RS05290	1,267	*Fe-hydrogenase beta subunit*
CTN_RS00355	1,267	*manganese-dependent inorganic pyrophosphatase*
CTN_RS06420	1,266	*Aldo/keto reductase*
CTN_RS00070	1,264	*Clostripain-related protein*
CTN_RS00595	1,263	*Malate oxidoreductase*
CTN_RS05405	1,262	*Metal dependent phosphohydrolase*
CTN_RS07330	1,261	*Putative uncharacterized protein*
CTN_RS01330	1,260	*Response regulator*
CTN_RS03140	1,260	*2-dehydro-3-deoxyphosphogluconate aldolase*
CTN_RS04095	1,258	*UvrB/UvrC protein*
CTN_RS09580	1,258	*Acyl carrier protein*
CTN_RS02055	1,257	*tRNA modification GTPase trmE*
CTN_RS09545	1,255	*Putative uncharacterized protein*
CTN_RS02720	1,247	*Glucosamine--fructose-6-phosphate aminotransferase*
CTN_RS08420	1,247	*Thiamine pyrophosphokinase*
CTN_RS03660	1,245	*Putative uncharacterized protein*
CTN_RS03480	1,245	*Putative uncharacterized protein*
CTN_RS08535	1,243	*Putative uncharacterized protein*
CTN_RS05690	1,237	*Queuine tRNA-ribosyltransferase*
CTN_RS00380	1,237	*Radical SAM domain protein precursor*
CTN_RS03610	1,232	*Putative uncharacterized protein*
CTN_RS01820	1,232	*Putative uncharacterized protein*
CTN_RS05300	1,221	*Stage II sporulation E family protein*
CTN_RS03835	1,220	*Binding-protein-dependent transport systems inner membrane component*

CTN_RS07230	1,216	*Putative uncharacterized protein*
CTN_RS06905	1,213	*ABC transporter*
CTN_RS04455	1,211	*Radical SAM enzyme, Cfr family*
CTN_RS08310	1,207	*Flagellar biosynthetic protein fliP*
CTN_RS04865	1,206	*Putative metalloprotease*
CTN_RS08230	1,199	*Protein Homology*
CTN_RS02815	1,199	*Major facilitator superfamily MFS_1 precursor*
CTN_RS04125	1,198	*DNA-directed DNA polymerase I*
CTN_RS09185	1,196	*Metal dependent phosphohydrolase*
CTN_RS06205	1,196	*Hypothetical Protein*
CTN_RS03020	1,196	*Putative uncharacterized protein*
CTN_RS02730	1,196	*ATP-dependent Clp protease ATP-binding subunit clpX*
CTN_RS04630	1,193	*RNA methyltransferase, TrmH family, group 3*
CTN_RS01505	1,193	*Propanediol utilization protein*
CTN_RS01020	1,192	*DNA polymerase III subunit alpha*
CTN_RS06440	1,191	*Exporter of the RND superfamily-like protein*
CTN_RS04490	1,188	*Putative uncharacterized protein*
CTN_RS05605	1,187	*Rod shape-determining protein MreB*
CTN_RS03265	1,186	*Queuosine biosynthesis protein QueD*
CTN_RS08280	1,186	*periplasmic serine protease*
CTN_RS03570	1,186	*16S rRNA m(2)G 1207 methyltransferase*
CTN_RS03640	1,185	*Alpha-amylase*
CTN_RS07750	1,184	*FadR family transcriptional regulator*
CTN_RS01515	1,184	*ABC transporter related*
CTN_RS08850	1,174	*PHP domain-containing protein*
CTN_RS03630	1,165	*Putative uncharacterized protein*
CTN_RS03975	1,160	*Putative uncharacterized protein*
CTN_RS02020	1,159	*Acetate kinase*
CTN_RS06140	1,147	*Twitching motility protein*
CTN_RS03025	1,143	*ComE protein*
CTN_RS08260	1,143	*Iron-containing alcohol dehydrogenase*
CTN_RS00800	1,142	*Binding-protein-dependent transport systems inner membrane component precursor*
CTN_RS03550	1,141	*Putative uncharacterized protein precursor*

CTN_RS06595	1,140	*Aspartate aminotransferase*
CTN_RS02215	1,139	*Phospho-N-acetylmuramoyl-pentapeptide-transferase*
CTN_RS08985	1,138	*Translation initiation factor IF-2*
CTN_RS03260	1,137	*Hypothetical Protein*
CTN_RS00820	1,129	*Oligopeptide ABC transporter, ATP-binding protein*
CTN_RS04635	1,125	*NagD protein*
CTN_RS03620	1,106	*Sensor protein*
CTN_RS09020	1,093	*Ras superfamily GTP-binding protein YlqF*
CTN_RS00090	1,085	*sugar transporter*
CTN_RS08745	-1,109	*Transcriptional regulator, TetR family*
CTN_RS05595	-1,109	*flagellar basal-body rod protein FlgG*
CTN_RS02715	-1,110	*Fatty acid/phospholipid synthesis protein plsX*
CTN_RS05650	-1,115	*ApbE family lipoprotein*
CTN_RS00960	-1,121	*Nicotinic acid phosphoribosyltransferase-like protein*
CTN_RS00860	-1,128	*NAD-dependent deacetylase*
CTN_RS08890	-1,131	*Putative uncharacterized protein*
CTN_RS08590	-1,140	*Metal dependent phosphohydrolase*
CTN_RS08295	-1,146	*MazG protein*
CTN_RS08350	-1,153	*RNA polymerase, sigma 28 subunit, FliA/WhiG*
CTN_RS07435	-1,156	*LexA repressor*
CTN_RS04575	-1,156	*Holliday junction ATP-dependent DNA helicase ruvB*
CTN_RS00035	-1,158	*Putative uncharacterized protein*
CTN_RS01980	-1,160	*Aldose 1-epimerase*
CTN_RS01855	-1,160	*Oxidoreductase domain protein*
CTN_RS04870	-1,161	*Metal dependent phosphohydrolase precursor*
CTN_RS05475	-1,162	*Putative uncharacterized protein*
CTN_RS08585	-1,166	*Ribosome-binding factor A*
CTN_RS02030	-1,168	*Pyruvate,orthophosphate dikinase*
CTN_RS04080	-1,170	*Ribose-phosphate pyrophosphokinase*
CTN_RS08285	-1,173	*Ribonuclease*
CTN_RS07240	-1,178	*Flagellar protein FlaG protein*
CTN_RS05785	-1,179	*Transcriptional regulator, Fis family*
CTN_RS02950	-1,179	*Sugar ABC transporter, ATP-binding protein*

CTN_RS00485	-1,180	*Uncharacterized protein*
CTN_RS03010	-1,181	*Putative uncharacterized protein*
CTN_RS08950	-1,186	*radical SAM protein*
CTN_RS09225	-1,187	*Putative uncharacterized protein*
CTN_RS06195	-1,190	*Glutamyl-tRNA synthetase 1*
CTN_RS05470	-1,191	*Major facilitator superfamily MFS_1 precursor*
CTN_RS03435	-1,193	*Cyclase family protein*
CTN_RS02200	-1,193	*Putative uncharacterized protein*
CTN_RS04170	-1,195	*ATP synthase gamma chain*
CTN_RS03440	-1,202	*Transglutaminase domain protein*
CTN_RS08470	-1,203	*Pyruvateferredoxin 2-oxidoreductase-related protein*
CTN_RS01390	-1,205	*Efflux transporter, RND family, MFP subunit precursor*
CTN_RS07560	-1,206	*ABC transporter relatec*
CTN_RS06855	-1,209	*Arabinogalactan endo-1,4-beta-galactosidase precursor*
CTN_RS07555	-1,209	*Putative uncharacterized protein*
CTN_RS06965	-1,209	*PleD-related protein*
CTN_RS02310	-1,212	*Glycyl-tRNA synthetase alpha subunit*
CTN_RS09220	-1,213	*Putative uncharacterized protein*
CTN_RS07585	-1,214	*Endoglucanase*
CTN_RS06600	-1,214	*Phosphorylated carbohydrates phosphatase*
CTN_RS06150	-1,219	*Response regulator*
CTN_RS02605	-1,220	*Putative uncharacterized protein*
CTN_RS05260	-1,223	*FAD dependent oxidoreductase precursor*
CTN_RS00350	-1,225	*Rod shape-determining protein MreB*
CTN_RS09405	-1,227	*Ray-related protein*
CTN_RS00610	-1,228	*Tryptophan synthase beta chain 2*
CTN_RS09190	-1,231	*Putative uncharacterized protein*
CTN_RS04565	-1,234	*3-methyl-2-oxobutanoate hydroxymethyltransferase*
CTN_RS00705	-1,236	*Putative uncharacterized protein precursor*
CTN_RS08320	-1,238	*Flagellar biosynthesis protein FlhA*
CTN_RS06315	-1,238	*ABC transporter related precursor*
CTN_RS06460	-1,239	*6-phospho-beta-glucosidase bglT*
CTN_RS09180	-1,242	*Threonyl-tRNA synthetase*

CTN_RS00765	-1,243	*Phage SPO1 DNA polymerase-related protein*
CTN_RS01790	-1,243	*ribose ABC transporter, permease protein*
CTN_RS08680	-1,249	*Type II secretion system protein E*
CTN_RS03470	-1,250	*CRISPR-associated protein, Csx2 family*
CTN_RS00125	-1,251	*Hypothetical Protein*
CTN_RS03310	-1,251	*ROK family protein*
CTN_RS08545	-1,255	*50S ribosomal protein L9*
CTN_RS02570	-1,258	*Primosomal protein N*
CTN_RS05430	-1,261	*Putative uncharacterized protein*
CTN_RS07595	-1,262	*septum site-determining protein minC*
CTN_RS00660	-1,263	*Oligopeptide ABC transporter, periplasmic oligopeptide-binding protein*
CTN_RS03255	-1,263	*Dihydropteroate synthase*
CTN_RS00930	-1,264	*Protein translocase subunit secG*
CTN_RS07260	-1,264	*General secretion pathway protein D*
CTN_RS00710	-1,272	*ATP-dependent hsl protease ATP-binding subunit hslU*
CTN_RS08045	-1,272	*Putative uncharacterized protein*
CTN_RS09390	-1,274	*Flagellar biosynthesis protein FliZ*
CTN_RS03645	-1,276	*Putative uncharacterized protein*
CTN_RS03285	-1,276	*4Fe-4S ferredoxin iron-sulfur binding domain protein*
CTN_RS08410	-1,277	*Putative zinc metalloprotease*
CTN_RS09415	-1,279	*trigger factor*
CTN_RS09645	-1,280	*Transcriptional regulator, SARP family*
CTN_RS03430	-1,281	*Hypothetical Protein*
CTN_RS04450	-1,283	*UDP-N-acetylenolpyruvoylglucosamine reductase*
CTN_RS07920	-1,284	*Helicase domain protein*
CTN_RS07370	-1,284	*RNA methyltransferase, TrmA family*
CTN_RS05315	-1,288	*Myo-inositol-1-phosphate synthase-related protein*
CTN_RS04385	-1,288	*MATE efflux family protein*
CTN_RS03345	-1,290	*Beta-glucosidase*
CTN_RS01445	-1,291	*Putative fibronectin-binding protein*
CTN_RS07480	-1,292	*Sugar kinase*
CTN_RS08900	-1,296	*Putative uncharacterized protein*
CTN_RS02380	-1,297	*ABC-type nitrate/sulfonate/bicarbonate transport systems periplasmic components-like protein precursor*

CTN_RS04595	-1,300	*Phosphate transport system protein phoU*
CTN_RS03305	-1,300	*Binding-protein-dependent transport systems inner membrane component*
CTN_RS06510	-1,301	*Type IV pilin-related protein precursor*
CTN_RS02700	-1,302	*Putative uncharacterized protein*
CTN_RS04755	-1,306	*TPR repeat-containing protein*
CTN_RS04105	-1,306	*GTP-binding protein lepA*
CTN_RS08480	-1,306	*Cation diffusion facilitator family transporter*
CTN_RS03105	-1,307	*Oligopeptide ABC transporter, permease protein*
CTN_RS05645	-1,308	*Radical SAM domain protein*
CTN_RS02635	-1,311	*Holliday junction branch migration protein RuvA*
CTN_RS07375	-1,315	*Arginyl-tRNA synthetase*
CTN_RS06180	-1,316	*Inosine-5-monophosphate dehydrogenase-related protein*
CTN_RS04110	-1,318	*Putative uncharacterized protein*
CTN_RS07055	-1,318	*Pyruvate ferredoxin/flavodoxin oxidoreductase, beta subunit*
CTN_RS02975	-1,319	*GTP-binding protein Obg/CgtA*
CTN_RS07495	-1,321	*Putative uncharacterized protein*
CTN_RS02750	-1,321	*hypothetical protein*
CTN_RS03275	-1,325	*Hypothetical Protein*
CTN_RS07060	-1,327	*Pyruvate flavodoxin/ferredoxin oxidoreductase domain protein*
CTN_RS02395	-1,327	*DNA repair protein RadA*
CTN_RS07930	-1,327	*Putative uncharacterized protein*
CTN_RS02065	-1,327	*UvrABC system protein C*
CTN_RS06480	-1,330	*Cell division protein FtsA*
CTN_RS08360	-1,332	*Putative uncharacterized protein*
CTN_RS07475	-1,335	*Glycoside hydrolase family 2, sugar binding*
CTN_RS04220	-1,336	*Radical SAM domain protein*
CTN_RS00195	-1,339	*Polysaccharide biosynthesis protein*
CTN_RS08250	-1,340	*Carbohydrate kinase, YjeF related protein*
CTN_RS02385	-1,340	*NADP-reducing hydrogenase, subunit D*
CTN_RS05780	-1,344	*peptide chain release factor 2*
CTN_RS05435	-1,348	*Putative uncharacterized protein*
CTN_RS08255	-1,350	*Lytic transglycosylase, catalytic*
CTN_RS00840	-1,350	*Condensin subunit ScpB*

CTN_RS09200	-1,352	*Mannose-6-phosphate isomerase*
CTN_RS08725	-1,352	*PfkB domain protein*
CTN_RS08080	-1,352	*Ribose ABC transporter, permease protein*
CTN_RS00690	-1,355	*tRNA delta(2)-isopentenylpyrophosphate transferase*
CTN_RS01210	-1,356	*Auxin Efflux Carrier*
CTN_RS04725	-1,360	*Bifunctional protein folD*
CTN_RS06990	-1,361	*Flagellar protein FliS*
CTN_RS03540	-1,361	*CRISPR-associated protein, Cas2 family*
CTN_RS05685	-1,362	*Formimidoyltetrahydrofolate cyclodeaminase*
CTN_RS04475	-1,362	*DNA mismatch repair protein mutS*
CTN_RS02360	-1,363	*Hypoxanthine phosphoribosyltransferase*
CTN_RS03300	-1,363	*Binding-protein-dependent transport systems inner membrane component precursor*
CTN_RS01905	-1,363	*Short-chain dehydrogenase/reductase SDR precursor*
CTN_RS07590	-1,364	*Endoglucanase*
CTN_RS02845	-1,368	*metal transport system ATP-binding protein*
CTN_RS09000	-1,369	*PSP1 domain protein*
CTN_RS06390	-1,370	*ABC-2 type transporter*
CTN_RS05570	-1,370	*Mg-protoporphyrin IX monomethyl ester oxidative cyclase-related protein*
CTN_RS08540	-1,374	*RNAse Z*
CTN_RS05995	-1,377	*Unknown*
CTN_RS03100	-1,378	*Oligopeptide/dipeptide ABC transporter, ATPase subunit*
CTN_RS00745	-1,380	*Putative uncharacterized protein*
CTN_RS09385	-1,382	*Chemotaxis protein cheY*
CTN_RS05660	-1,383	*Heptaprenyl diphosphate synthase component I precursor*
CTN_RS02255	-1,383	*NADH-quinone oxidoreductase, E subunit*
CTN_RS02870	-1,383	*Acetamidase*
CTN_RS02010	-1,385	*L-arabinose isomerase*
CTN_RS05665	-1,385	*Maf-like protein*
CTN_RS01945	-1,386	*6-phosphofructokinase, pyrophosphate-dependent*
CTN_RS09500	-1,386	*Flagellar protein*
CTN_RS09420	-1,386	*Putative uncharacterized protein*
CTN_RS08075	-1,387	*Ribose import ATP-binding protein rbsA 2*
CTN_RS09490	-1,388	*CheC, inhibitor of MCP methylation*

CTN_RS06605	-1,391	*glutamine-dependent NAD(+) synthetase*
CTN_RS06115	-1,392	*Putative uncharacterized protein*
CTN_RS02250	-1,397	*NADP-reducing hydrogenase, subunit C*
CTN_RS03360	-1,400	*DNA mismatch repair protein mutL*
CTN_RS07655	-1,401	*Putative UDP-N-acetylglucosamine 2-epimerase*
CTN_RS08630	-1,401	*GTPase Era*
CTN_RS01150	-1,401	*6-phosphogluconate dehydrogenase, decarboxylating*
CTN_RS08165	-1,401	*Aspartate 1-decarboxylase precursor*
CTN_RS04335	-1,403	*YicC N-terminal domain protein*
CTN_RS00845	-1,403	*Putative uncharacterized protein*
CTN_RS09450	-1,404	*Glyceraldehyde-3-phosphate dehydrogenase*
CTN_RS00940	-1,407	*Outer membrane protein alpha precursor*
CTN_RS04580	-1,410	*Alanine racemase domain protein*
CTN_RS03750	-1,411	*Alpha-glucosidase*
CTN_RS08315	-1,413	*Flagellar biosynthesis protein FlhE*
CTN_RS04425	-1,416	*Hypothetical Protein*
CTN_RS04115	-1,417	*Glycerophosphoryl diester phosphodiesterase*
CTN_RS03825	-1,417	*Periplasmic binding protein/LacI transcriptional regulator precursor*
CTN_RS00935	-1,425	*Tyrosyl-tRNA synthetase*
CTN_RS03465	-1,428	*CRISPR-associated protein, Cas6 family*
CTN_RS07760	-1,428	*VWA domain-containing protein*
CTN_RS08595	-1,437	*Histidine kinase precursor*
CTN_RS02600	-1,438	*Adenosylhomocysteinase*
CTN_RS06270	-1,440	*Helix-turn-helix domain protein*
CTN_RS05230	-1,445	*glycoside hydrolase*
CTN_RS02470	-1,448	*Stage V sporulation protein S*
CTN_RS05845	-1,449	*Putative uncharacterized protein*
CTN_RS00665	-1,449	*Oligopeptide ABC transporter, ATP-binding protein*
CTN_RS03690	-1,452	*Valyl-tRNA synthetase*
CTN_RS00670	-1,452	*Heavy metal binding protein*
CTN_RS05775	-1,453	*Protein translocase subunit secA*
CTN_RS02925	-1,457	*UDP-N-acetylglucosamine 1-carboxyvinyltransferase*
CTN_RS01630	-1,461	*Radical SAM N-terminal domain protein*

CTN_RS02985	-1,463	*TIM-barrel protein, nifR3 family*
CTN_RS08100	-1,464	*Putative uncharacterized protein*
CTN_RS02130	-1,466	*Glutamyl-tRNA(Gln) amidotransferase subunit C*
CTN_RS08815	-1,467	*Binding-protein-dependent transport systems inner membrane component*
CTN_RS04340	-1,471	*Aminotransferase, class V*
CTN_RS06395	-1,473	*ABC-2 type transporter*
CTN_RS02035	-1,473	*Putative uncharacterized protein*
CTN_RS08820	-1,473	*Extracellular solute-binding protein, family 1 precursor*
CTN_RS09335	-1,474	*Transcriptional regulator, MarR family*
CTN_RS02540	-1,477	*Putative uncharacterized protein*
CTN_RS03095	-1,480	*Oligopeptide ABC transporter, ATP-binding protein*
CTN_RS09355	-1,480	*Putative uncharacterized protein*
CTN_RS03415	-1,481	*NADP-reducing hydrogenase, subunit E*
CTN_RS05215	-1,481	*aspartate--tRNA ligase*
CTN_RS02315	-1,488	*Protein synthesis inhibitor*
CTN_RS05085	-1,488	*GTP-binding protein engB*
CTN_RS00685	-1,489	*Protein hfq*
CTN_RS04010	-1,490	*bifunctional aspartate carbamoyltransferase catalytic/regulatory subunit*
CTN_RS05670	-1,492	*DNA repair protein radC like protein*
CTN_RS02885	-1,497	*Sugar kinase, FGGY family*
CTN_RS03350	-1,500	*Laminarinase*
CTN_RS00775	-1,503	*UDP-glucose 4-epimerase*
CTN_RS02120	-1,506	*SsrA-binding protein*
CTN_RS08685	-1,510	*Cell division protein ftsZ*
CTN_RS07675	-1,513	*Transcriptional regulator, TetR family*
CTN_RS02175	-1,514	*Radical SAM domain protein*
CTN_RS01970	-1,517	*Sugar kinase, FGGY family*
CTN_RS09495	-1,520	*Flagellar motor switch protein FliM*
CTN_RS08990	-1,524	*Ribosomal protein L7Ae/L30e/S12e/Gadd45*
CTN_RS04730	-1,528	*Exodeoxyribonuclease 7 large subunit*
CTN_RS01755	-1,529	*Putative uncharacterized protein*
CTN_RS07830	-1,530	*ATPase*
CTN_RS01415	-1,532	*Diacylglycerol kinase catalytic region*

CTN_RS06995	-1,533	*Acetyltransferase-related protein*
CTN_RS09505	-1,537	*Motility protein E*
CTN_RS02535	-1,542	*Putative uncharacterized protein*
CTN_RS07545	-1,542	*Potassium channel*
CTN_RS00700	-1,543	*Heat shock protein 70kD*
CTN_RS08325	-1,544	*Flagellar biosynthesis protein FlhF*
CTN_RS03830	-1,546	*Binding-protein-dependent transport systems inner membrane component precursor*
CTN_RS05425	-1,546	*Putative metal dependent phosphohydrolase precursor*
CTN_RS01885	-1,546	*Alpha-L-fucosidase*
CTN_RS06715	-1,549	*Glycosyl transferase, group 1*
CTN_RS09135	-1,549	*sporulation protein*
CTN_RS04750	-1,549	*Dak phosphatase*
CTN_RS04350	-1,550	*thiamine biosynthesis protein thiI*
CTN_RS05975	-1,552	*ATP-dependent Clp protease, ATPase subunit*
CTN_RS01400	-1,553	*Outer membrane protein-like protein precursor*
CTN_RS04065	-1,553	*Putative uncharacterized protein*
CTN_RS04035	-1,557	*Putative uncharacterized protein*
CTN_RS01860	-1,558	*Putative uncharacterized protein*
CTN_RS01775	-1,566	*Ureidoglycolate hydrolase*
CTN_RS03770	-1,570	*Maltose ABC transporter, periplasmic maltose-binding protein*
CTN_RS07915	-1,572	*Putative uncharacterized protein*
CTN_RS03055	-1,573	*Flagellar hook-associated protein 3*
CTN_RS05620	-1,574	*Metal dependent phosphohydrolase*
CTN_RS04430	-1,576	*Hypothetical Protein*
CTN_RS00180	-1,579	*Putative repeat unit transporter*
CTN_RS06880	-1,579	*Extracellular solute-binding protein family 5 precursor*
CTN_RS00390	-1,580	*Cell division protein FtsH*
CTN_RS02280	-1,581	*Putative ABC transporter ATP-binding protein*
CTN_RS04100	-1,581	*Beta-mannosidase Man2*
CTN_RS09215	-1,581	*Sigma-B regulator*
CTN_RS08740	-1,582	*AstB/chuR-related protein*
CTN_RS09395	-1,583	*Flagellar biosynthesis protein FliF*
CTN_RS06155	-1,585	*Sensor protein*

CTN_RS02300	-1,585	*Flagellum-specific ATP synthase*
CTN_RS05375	-1,585	*Uncharacterized protein*
CTN_RS05655	-1,585	*Putative uncharacterized protein precursor*
CTN_RS04420	-1,586	*Putative uncharacterized protein*
CTN_RS04070	-1,589	*Putative uncharacterized protein*
CTN_RS01975	-1,590	*Sugar isomerase*
CTN_RS07000	-1,593	*HAD-superfamily hydrolase, subfamily IA, variant 1*
CTN_RS04745	-1,593	*Putative uncharacterized protein*
CTN_RS00440	-1,600	*Heat shock serine protease, periplasmic*
CTN_RS08090	-1,600	*transketolase, C-terminal subunit*
CTN_RS00850	-1,600	*Tryptophanyl-tRNA synthetase*
CTN_RS08160	-1,603	*Pseudouridine synthase*
CTN_RS03060	-1,604	*Flagellar assembly factor fliW*
CTN_RS04610	-1,605	*Inosine guanosine and xanthosine phosphorylase family*
CTN_RS08490	-1,607	*Peptidase M22, glycoprotease*
CTN_RS03120	-1,609	*Endo-1,4-beta-xylanase B precursor*
CTN_RS06030	-1,610	*NUDIX hydrolase*
CTN_RS02505	-1,611	*Response regulator*
CTN_RS03490	-1,611	*DnaJ-related protein*
CTN_RS02400	-1,613	*ATP-dependent Clp protease, ATPase subunit*
CTN_RS01550	-1,616	*endonuclease III domain-containing protein*
CTN_RS07485	-1,618	*Sugar-phosphate aldolase*
CTN_RS03090	-1,618	*Endo-1,3-beta-xylanase*
CTN_RS04040	-1,619	*DNA double-strand break repair rad50 ATPase*
CTN_RS09070	-1,623	*Lipopolysaccharide biosynthesis protein*
CTN_RS06710	-1,628	*Alpha-mannosidase-related protein*
CTN_RS08435	-1,629	*Putative uncharacterized protein*
CTN_RS02920	-1,629	*Pyruvate formate lyase activating enzyme*
CTN_RS08055	-1,631	*Ribokinase*
CTN_RS06295	-1,632	*AstB/chuR-related protein*
CTN_RS06305	-1,638	*Putative uncharacterized protein*
CTN_RS04165	-1,639	*ATP synthase subunit alpha*
CTN_RS04380	-1,640	*HAD-superfamily hydrolase, subfamily IA, variant 1*

CTN_RS05640	-1,644	*Hypothetical Protein*
CTN_RS08070	-1,645	*Putative uncharacterized protein*
CTN_RS08635	-1,652	*cytidine deaminase*
CTN_RS02210	-1,653	*UDP-N-acetylmuramoyl-tripeptide--D-alanyl-D-alanine ligase*
CTN_RS01670	-1,653	*Putative uncharacterized protein*
CTN_RS00080	-1,654	*Putative uncharacterized protein*
CTN_RS07815	-1,654	*Putative uncharacterized protein*
CTN_RS08290	-1,655	*Hypothetical Protein*
CTN_RS01890	-1,657	*Fructose-bisphosphate aldolase*
CTN_RS09195	-1,661	*Uncharacterized protein*
CTN_RS07465	-1,661	*Phosphodiesterase, MJ0936 family*
CTN_RS01965	-1,664	*AraM protein*
CTN_RS01155	-1,666	*TRAP dicarboxylate transporter, DctM subunit*
CTN_RS02290	-1,667	*Flagellar motor switch protein fliG*
CTN_RS08995	-1,667	*Putative uncharacterized protein*
CTN_RS05400	-1,674	*Lipopolysaccharide biosynthesis protein-related protein*
CTN_RS05970	-1,683	*Putative uncharacterized protein*
CTN_RS07660	-1,686	*Mannose-1-phosphate guanylyltransferase*
CTN_RS02860	-1,690	*L-lysine 2,3-aminomutase*
CTN_RS09240	-1,690	*Putative uncharacterized protein*
CTN_RS05500	-1,692	*Putative uncharacterized protein*
CTN_RS02930	-1,693	*putative diguanylate cyclase*
CTN_RS06185	-1,699	*Putative uncharacterized protein*
CTN_RS00230	-1,703	*Putative uncharacterized protein*
CTN_RS02270	-1,703	*Biotin--(Acetyl-CoA carboxylase) synthetase*
CTN_RS01395	-1,706	*Outer membrane protein-like protein precursor*
CTN_RS08330	-1,708	*Cobyrinic acid a,c-diamide synthase*
CTN_RS02960	-1,709	*Putative uncharacterized protein*
CTN_RS09340	-1,710	*Hypothetical Protein*
CTN_RS05935	-1,718	*Ribosome recycling factor*
CTN_RS09160	-1,718	*1,2-diacylglycerol 3-glucosyltransferase*
CTN_RS09400	-1,718	*Flagellar biosynthetic protein FliG*
CTN_RS05625	-1,719	*Lipopolysaccharide biosynthesis protein*

CTN_RS09640	-1,726	*Putative uncharacterized protein*
CTN_RS07385	-1,726	*Putative uncharacterized protein*
CTN_RS02630	-1,727	*Folylpolyglutamate synthase/dihydrofolate synthase*
CTN_RS08335	-1,728	*Type IV pilus assembly PilZ*
CTN_RS07835	-1,731	*Cupin 2, conserved barrel domain protein*
CTN_RS02465	-1,734	*CTN_trnaArg3*
CTN_RS02320	-1,735	*glycine dehydrogenase subunit 2*
CTN_RS06445	-1,736	*Methyltransferase type 11*
CTN_RS00620	-1,738	*Tryptophan synthase beta chain 2*
CTN_RS00060	-1,740	*NH(3)-dependent NAD(+) synthetase*
CTN_RS06980	-1,740	*NUDIX hydrolase*
CTN_RS07740	-1,742	*Alpha-glucosidase*
CTN_RS09235	-1,744	*(P)ppGpp synthetase*
CTN_RS07975	-1,747	*DsrH family protein*
CTN_RS00770	-1,748	*Iron-dependent transcriptional repressor*
CTN_RS03225	-1,748	*Iron-sulfur cluster-binding protein*
CTN_RS02285	-1,749	*Flagellar M-ring protein*
CTN_RS09510	-1,756	*Motility protein A*
CTN_RS09380	-1,760	*Chemotaxis protein cheW*
CTN_RS06375	-1,761	*ABC transporter, ATP-binding protein*
CTN_RS06035	-1,763	*Putative uncharacterized protein*
CTN_RS03845	-1,765	*Alpha amylase, catalytic region precursor*
CTN_RS02125	-1,765	*Hypothetical Protein*
CTN_RS05370	-1,774	*Lipopolysaccharide biosynthesis protein-related protein*
CTN_RS03485	-1,778	*Putative uncharacterized protein*
CTN_RS00135	-1,778	*hypothetical protein*
CTN_RS07770	-1,780	*Putative uncharacterized protein precursor*
CTN_RS01000	-1,781	*Hypothetical Protein*
CTN_RS05235	-1,781	*Dimethyladenosine transferase*
CTN_RS02355	-1,782	*Zn-dependent hydrolase of the beta-lactamase fold-like protein*
CTN_RS02345	-1,788	*6-phosphofructokinase*
CTN_RS07880	-1,794	*Putative uncharacterized protein*
CTN_RS03230	-1,796	*Putative uncharacterized protein*

CTN_RS05205	-1,797	*cytidylate kinase*
CTN_RS05080	-1,799	*Metal dependent phosphohydrolase*
CTN_RS06020	-1,799	*Adenine phosphoribosyltransferase*
CTN_RS02350	-1,800	*Pyruvate kinase*
CTN_RS02375	-1,801	*ABC transporter, permease protein, cysTW family*
CTN_RS03560	-1,803	*Succinyl-diaminopimelate desuccinylase*
CTN_RS02205	-1,803	*UDP-N-acetylmuramoyl-L-alanyl-D-glutamate--LD-lysine ligase*
CTN_RS02075	-1,812	*tRNA uridine 5-carboxymethylaminomethyl modification enzyme gidA*
CTN_RS00600	-1,813	*Fumarate hydratase, C-terminal subunit*
CTN_RS02655	-1,816	*Geranyltranstransferase*
CTN_RS02980	-1,816	*nicotinate (nicotinamide) nucleotide adenylyltransferase*
CTN_RS08060	-1,817	*Ribose ABC transporter, membrane-associated protein*
CTN_RS08065	-1,817	*Ribose ABC transporter, periplasmic ribose-binding protein*
CTN_RS05075	-1,820	*hypothetical protein*
CTN_RS03790	-1,824	*Putative uncharacterized protein precursor*
CTN_RS01760	-1,828	*Putative sugar ABC transporter, permease component*
CTN_RS06120	-1,835	*Flagellar hook-basal body complex protein fliE*
CTN_RS07335	-1,838	*Ribonuclease 3*
CTN_RS06850	-1,839	*Maltose ABC transporter, permease protein*
CTN_RS09245	-1,840	*PmbA-related protein*
CTN_RS07500	-1,846	*Transcriptional regulator, DeoR family*
CTN_RS02530	-1,847	*Putative uncharacterized protein*
CTN_RS01570	-1,854	*NAD(P)H-dependent glycerol-3-phosphate dehydrogenase*
CTN_RS09155	-1,855	*Putative uncharacterized protein*
CTN_RS04255	-1,856	*Diguanylate cyclase and serine/threonine protein kinase with TPR repeats*
CTN_RS01170	-1,856	*ABC transporter related*
CTN_RS08095	-1,860	*Glycerol kinase 1*
CTN_RS06385	-1,864	*Putative uncharacterized protein*
CTN_RS07160	-1,865	*CBS domain containing protein*
CTN_RS03760	-1,866	*Maltose ABC transporter, permease protein*
CTN_RS03250	-1,873	*2-amino-4-hydroxy-6-hydroxymethyldihydropteridine pyrophosphokinase*
CTN_RS05165	-1,873	*RNA polymerase sigma factor rpoD*
CTN_RS06955	-1,879	*Regulatory protein, FmdB family*

CTN_RS08110	-1,886	*Transcriptional regulator, LacI family*
CTN_RS01195	-1,887	*Binding-protein-dependent transport systems inner membrane component precursor*
CTN_RS05790	-1,891	*Type I phosphodiesterase/nucleotide pyrophosphatase*
CTN_RS01165	-1,892	*C4-dicarboxylate-binding protein*
CTN_RS04445	-1,895	*Proline dipeptidase*
CTN_RS08195	-1,895	*Putative uncharacterized protein*
CTN_RS07430	-1,898	*Putative uncharacterized protein*
CTN_RS04605	-1,904	*Primary replicative DNA helicase*
CTN_RS07680	-1,906	*ABC transporter, permease protein*
CTN_RS04655	-1,906	*butyrate kinase 1*
CTN_RS05615	-1,907	*Single stranded DNA-specific exonuclease*
CTN_RS06300	-1,910	*AstB/chuR-related protein*
CTN_RS00720	-1,913	*tRNA (5-methylaminomethyl-2-thiouridylate)-methyltransferase*
CTN_RS09005	-1,916	*DNA polymerase III, gamma subunit-related protein*
CTN_RS06865	-1,923	*Extracellular solute-binding protein family 1*
CTN_RS08210	-1,924	*ATPase associated with various cellular activities, AAA_5*
CTN_RS06475	-1,925	*MutS2 protein*
CTN_RS08050	-1,926	*LemA family protein precursor*
CTN_RS04060	-1,928	*Putative uncharacterized protein*
CTN_RS04345	-1,928	*1-acyl-sn-glycerol-3-phosphate acetyltransferase*
CTN_RS08155	-1,930	*Putative uncharacterized protein*
CTN_RS00150	-1,931	*Glycosyl transferase, family 2*
CTN_RS07935	-1,937	*Putative uncharacterized protein*
CTN_RS01795	-1,938	*Putative uncharacterized protein*
CTN_RS05815	-1,951	*hypothetical protein*
CTN_RS05750	-1,962	*Pyrrolo-quinoline quinone precursor*
CTN_RS06025	-1,964	*4-diphosphocytidyl-2-C-methyl-D-erythritol kinase*
CTN_RS03005	-1,966	*Putative uncharacterized protein precursor*
CTN_RS02995	-1,967	*General secretion pathway protein F*
CTN_RS09085	-1,973	*Flagellin*
CTN_RS03355	-1,975	*Methyl-accepting chemotaxis protein*
CTN_RS02510	-1,977	*Activator of Hsp90 ATPase 1 family protein*
CTN_RS05810	-1,984	*Glycerate kinase*

CTN_RS06320	-1,986	*Fe-S oxidoreductase-like protein*
CTN_RS04045	-1,993	*Exonuclease*
CTN_RS04140	-1,995	*F-ATPase I-subunit*
CTN_RS03940	-2,002	*Endonuclease V*
CTN_RS03775	-2,004	*Alpha-amylase*
CTN_RS03765	-2,004	*Binding-protein-dependent transport systems inner membrane component precursor*
CTN_RS01300	-2,009	*2-oxoglutarate ferredoxin oxidoreductase, beta subunit*
CTN_RS00730	-2,009	*Putative uncharacterized protein*
CTN_RS03945	-2,012	*Membrane bound protein LytF*
CTN_RS09175	-2,023	*Phosphopantetheine adenylyltransferase*
CTN_RS02990	-2,029	*Rubrerythrin*
CTN_RS02915	-2,030	*ROK family protein*
CTN_RS03635	-2,030	*Elongation factor G-like protein*
CTN_RS04875	-2,035	*PhoH-related protein*
CTN_RS04695	-2,043	*UvrABC system protein B*
CTN_RS09205	-2,043	*Putative uncharacterized protein*
CTN_RS07820	-2,047	*Putative uncharacterized protein*
CTN_RS01815	-2,056	*Monosaccharide-transporting ATPase*
CTN_RS08425	-2,057	*methylated-DNA--[protein]-cysteine S-methyltransferase*
CTN_RS01185	-2,064	*Beta-lactamase domain protein*
CTN_RS00175	-2,067	*Putative dTDP-4-dehydrorhamnose reductase*
CTN_RS05840	-2,069	*Unknown*
CTN_RS02890	-2,070	*Ribose import ATP-binding protein rbsA 1*
CTN_RS07970	-2,074	*Uncharacterized protein involved in the oxidation of intracellular sulfur-like protein*
CTN_RS05365	-2,075	*hypothetical protein*
CTN_RS04325	-2,077	*Guanylate kinase*
CTN_RS02180	-2,088	*Putative uncharacterized protein*
CTN_RS01190	-2,091	*ABC-type, ATP binding, spermidine/spermine transporter, PotA*
CTN_RS08735	-2,096	*AstB/chuR-related protein*
CTN_RS06950	-2,100	*Hypothetical Protein*
CTN_RS02185	-2,105	*Putative uncharacterized protein*
CTN_RS06845	-2,106	*Maltose ABC transporter, permease protein*
CTN_RS00155	-2,106	*Glucose-1-phosphate thymidylyltransferase*

CTN_RS08430	-2,107	*Penicillin-binding protein, class 1A*
CTN_RS09555	-2,108	*Hydrolase, TatD family*
CTN_RS06380	-2,113	*ABC-2 type transporter*
CTN_RS07690	-2,115	*Sugarcation symporter family protein*
CTN_RS03785	-2,117	*Putative uncharacterized protein*
CTN_RS02705	-2,124	*Putative uncharacterized protein*
CTN_RS05480	-2,124	*Radical SAM domain protein*
CTN_RS03810	-2,125	*ROK family protein*
CTN_RS03295	-2,127	*Extracellular solute-binding protein, family 1 precursor*
CTN_RS06470	-2,136	*Putative uncharacterized protein*
CTN_RS04310	-2,140	*ErfK/YbiS/YcfS/YnhG family protein*
CTN_RS06190	-2,145	*PHP C-terminal domain protein*
CTN_RS02295	-2,145	*Flagellar export/assembly protein*
CTN_RS03340	-2,149	*Putative uncharacterized protein*
CTN_RS01440	-2,153	*endonuclease 4*
CTN_RS09250	-2,155	*TldD protein*
CTN_RS05240	-2,158	*antiterminator*
CTN_RS06165	-2,160	*Putative uncharacterized protein*
CTN_RS01320	-2,172	*Thymidine kinase*
CTN_RS07600	-2,180	*Alpha/beta hydrolase fold protein*
CTN_RS00955	-2,183	*Pyrazinamidase/nicotinamidase-related protein*
CTN_RS03755	-2,188	*Cyclomaltodextrinase glucanotransferase*
CTN_RS00225	-2,189	*ATPase-like protein*
CTN_RS03815	-2,189	*ABC transporter related*
CTN_RS07810	-2,193	*Glutamate dehydrogenase*
CTN_RS05490	-2,209	*Putative uncharacterized protein*
CTN_RS00310	-2,213	*Sugar ABC transporter, periplasmic sugar-binding protein*
CTN_RS02240	-2,215	*Phosphodiesterase, MJ0936 family*
CTN_RS06340	-2,215	*ABC transporter related precursor*
CTN_RS07040	-2,218	*Alpha-glucan phosphorylase*
CTN_RS02905	-2,220	*Sugar ABC transporter, permease protein*
CTN_RS06975	-2,225	*Condensin subunit Smc*
CTN_RS06040	-2,226	*Putative uncharacterized protein*

CTN_RS06260	-2,235	*hypothetical protein*
CTN_RS00395	-2,237	*tRNA(Ile)-lysidine synthase*
CTN_RS07685	-2,240	*ABC transporter*
CTN_RS04675	-2,241	*2-oxoisovalerate oxidoreductase, beta subunit*
CTN_RS03000	-2,249	*Putative uncharacterized protein*
CTN_RS07215	-2,256	*MTA/SAH nucleosidase*
CTN_RS08200	-2,263	*Restriction endonuclease*
CTN_RS02080	-2,271	*DNA polymerase III, beta subunit*
CTN_RS02575	-2,289	*Putative uncharacterized protein*
CTN_RS03315	-2,290	*Oligopeptide ABC transporter, periplasmic oligopeptide-binding protein*
CTN_RS00050	-2,292	*2-C-methyl-D-erythritol 2,4-cycloa*
CTN_RS00190	-2,296	*Glycosyl transferase group 1*
CTN_RS06045	-2,299	*Seryl-tRNA synthetase*
CTN_RS09140	-2,300	*tRNA methyltransferase complex GCD14 subunit precursor*
CTN_RS08040	-2,312	*Oligoendopeptidase, M3 family*
CTN_RS00045	-2,316	*Putative uncharacterized protein*
CTN_RS09550	-2,319	*Pleiotropic regulatory protein*
CTN_RS04440	-2,324	*Putative uncharacterized protein*
CTN_RS01805	-2,327	*oxidoreductase, short chain dehydrogenase/reductase family*
CTN_RS05360	-2,340	*Putative uncharacterized protein*
CTN_RS00140	-2,345	*Glycosyl transferase, group 1*
CTN_RS05965	-2,348	*2-C-methyl-D-erythritol 4-phosphate cytidylyltransferase*
CTN_RS06080	-2,350	*Putative uncharacterized protein*
CTN_RS01895	-2,351	*Carbohydrate kinase, FGGY*
CTN_RS03495	-2,392	*Putative uncharacterized protein*
CTN_RS01435	-2,395	*Putative uncharacterized protein*
CTN_RS04740	-2,396	*1-deoxy-D-xylulose-5-phosphate synthase*
CTN_RS08475	-2,400	*phosphopyruvate hydratase*
CTN_RS02325	-2,400	*glycine dehydrogenase subunit 1*
CTN_RS01780	-2,408	*Monosaccharide-transporting ATPase precursor*
CTN_RS07940	-2,423	*hypothetical protein*
CTN_RS06170	-2,434	*Positive regulator of sigma E, RseC/MucC*
CTN_RS00945	-2,439	*Putative uncharacterized protein*

CTN_RS06175	-2,444	*DRTGG domain protein*
CTN_RS07550	-2,446	*Divalent-cation tolerance protein cutA*
CTN_RS05800	-2,448	*Putative uncharacterized protein*
CTN_RS07695	-2,449	*Extracellular solute-binding protein, family 1 precursor*
CTN_RS09230	-2,470	*D-tyrosyl-tRNA(Tyr) deacylase*
CTN_RS06725	-2,473	*Transcriptional regulator, RpiR family*
CTN_RS02680	-2,483	*Flavin reductase domain protein, FMN-binding*
CTN_RS03565	-2,487	*Histidine kinase*
CTN_RS04355	-2,487	*Tetratricopeptide TPR_2 repeat protein*
CTN_RS09485	-2,495	*Dehydrase-related protein*
CTN_RS09375	-2,495	*Chemotaxis protein cheA*
CTN_RS05220	-2,506	*Translation initiation factor, eIF-2B alpha subunit-related*
CTN_RS01770	-2,508	*Extracellular solute-binding protein family 1 precursor*
CTN_RS06465	-2,509	*ATPase, BadF/BadG/BcrA/BcrD type*
CTN_RS02955	-2,518	*Basic membrane protein*
CTN_RS05765	-2,520	*Hemolysin A precursor*
CTN_RS05210	-2,524	*Putative anti-sigma factor antagonist*
CTN_RS04480	-2,527	*GCN5-related N-acetyltransferase*
CTN_RS02340	-2,530	*Putative uncharacterized protein*
CTN_RS04435	-2,532	*Putative uncharacterized protein*
CTN_RS08340	-2,537	*CheY-P phosphatase cheC*
CTN_RS00215	-2,538	*HEPN domain-containing protein*
CTN_RS00605	-2,545	*Fumarate hydratase, N-terminal subunit*
CTN_RS00760	-2,545	*Flagellar export protein FliJ*
CTN_RS03400	-2,562	*Methyl-accepting chemotaxis sensory transducer*
CTN_RS08205	-2,567	*Putative uncharacterized protein*
CTN_RS08655	-2,568	*Response regulator*
CTN_RS02190	-2,571	*Glucose-1-phosphate adenylyltransferase*
CTN_RS00235	-2,571	*Chromosome segregation ATPase-like protein precursor*
CTN_RS00185	-2,576	*Hypothetical Protein*
CTN_RS07865	-2,582	*Transposase*
CTN_RS00415	-2,584	*DNA polymerase III polC-type*
CTN_RS06970	-2,585	*Oxidoreductase, aldo/keto reductase family*

CTN_RS01995	-2,586	*Binding-protein-dependent transport systems inner membrane component*
CTN_RS04250	-2,591	*Phosphoesterase, RecJ domain protein*
CTN_RS00735	-2,592	*Putative uncharacterized protein*
CTN_RS03840	-2,596	*Extracellular solute-binding protein, family 1 precursor*
CTN_RS03500	-2,600	*CRISPR-associated protein, Cas6 family*
CTN_RS04390	-2,602	*Putative uncharacterized protein*
CTN_RS02550	-2,616	*23S ribosomal RNA*
CTN_RS02195	-2,618	*Glucose-1-phosphate adenylyltransferase, GlgD subunit*
CTN_RS09145	-2,618	*Carboxyl-terminal protease precursor*
CTN_RS01960	-2,620	*Transcriptional regulator, PadR family*
CTN_RS04665	-2,623	*butyrate kinase 2*
CTN_RS08650	-2,631	*Formiminotransferase-cyclodeaminase/formiminotetrahydrofolate cyclodeaminase*
CTN_RS06400	-2,632	*ABC transporter related*
CTN_RS03535	-2,632	*CRISPR-associated protein, Cas1 family*
CTN_RS09345	-2,632	*Biotin/lipoate A/B protein ligase*
CTN_RS04225	-2,633	*Putative uncharacterized protein*
CTN_RS02820	-2,644	*Carboxypeptidase G2*
CTN_RS05590	-2,644	*Flagellar protein FlgA*
CTN_RS04315	-2,649	*DNA/pantothenate metabolism flavoprotein*
CTN_RS01305	-2,649	*Deoxycytidylate deaminase*
CTN_RS02660	-2,654	*Putative uncharacterized protein*
CTN_RS08085	-2,661	*transketolase, N-terminal subunit*
CTN_RS07755	-2,671	*hypothetical protein*
CTN_RS00170	-2,672	*Putative uncharacterized protein*
CTN_RS07965	-2,673	*DsrE family protein*
CTN_RS04050	-2,675	*Putative uncharacterized protein*
CTN_RS05770	-2,679	*Putative uncharacterized protein precursor*
CTN_RS09350	-2,687	*Methyltransferase gidB*
CTN_RS02000	-2,695	*Binding-protein-dependent transport systems inner membrane component*
CTN_RS02970	-2,703	*Putative uncharacterized protein*
CTN_RS07030	-2,705	*Metal dependent phosphohydrolase*
CTN_RS08620	-2,723	*Chaperone protein dnaJ*
CTN_RS07035	-2,730	*Short-chain dehydrogenase/reductase SDR*

CTN_RS01490	-2,730	*Acriflavin resistance protein*
CTN_RS06515	-2,732	*Endo-1,4-beta-xylanase precursor*
CTN_RS09150	-2,753	*Putative uncharacterized protein*
CTN_RS04235	-2,756	*RNA polymerase sigma-E factor*
CTN_RS06705	-2,765	*ABC transporter related*
CTN_RS03855	-2,766	*Cellobiose-phosphorylase*
CTN_RS06405	-2,772	*Radical SAM domain protein*
CTN_RS06160	-2,774	*Putative uncharacterized protein*
CTN_RS02790	-2,775	*Radical SAM domain protein*
CTN_RS06130	-2,780	*Flagellar basal-body rod protein FlgE*
CTN_RS02330	-2,786	*Glycine cleavage system H protein*
CTN_RS09095	-2,791	*end_range=1786187,.*
CTN_RS00295	-2,793	*PHP C-terminal domain protein precursor*
CTN_RS04680	-2,796	*Thiamine pyrophosphate enzyme domain protein TPP-binding*
CTN_RS04705	-2,797	*Elongation factor P*
CTN_RS02005	-2,806	*Extracellular solute-binding protein family 1 precursor*
CTN_RS05675	-2,817	*Metal dependent phosphohydrolase*
CTN_RS02625	-2,823	*Phosphopentomutase*
CTN_RS00300	-2,828	*Monosaccharide-transporting ATPase precursor*
CTN_RS09075	-2,834	*Putative uncharacterized protein*
CTN_RS01175	-2,872	*Inner-membrane translocator*
CTN_RS07785	-2,876	*Major facilitator superfamily MFS_1*
CTN_RS01810	-2,882	*Putative ribose/galactose/methyl galactoside import ATP-binding protein*
CTN_RS02545	-2,888	*5S ribosomal RNA*
CTN_RS07735	-2,893	*Putative uncharacterized protein*
CTN_RS07955	-2,894	*Hypothetical Protein*
CTN_RS07780	-2,898	*Esterase*
CTN_RS08000	-2,913	*Methyl-accepting chemoreceptor-related protein*
CTN_RS01990	-2,915	*Putative uncharacterized protein*
CTN_RS06410	-2,938	*hypothetical protein*
CTN_RS01295	-2,947	*2-oxoglutarate ferredoxin oxidoreductase, gamma subunit*
CTN_RS03405	-2,949	*Beta-lactamase domain protein*
CTN_RS09255	-2,949	*Putative uncharacterized protein*

CTN_RS04245	-2,977	*purine-nucleoside phosphorylase*
CTN_RS02410	-2,980	*protein of unknown function DUF355*
CTN_RS02335	-2,982	*Aminomethyltransferase*
CTN_RS01410	-3,002	*Putative uncharacterized protein*
CTN_RS01200	-3,002	*Binding-protein-dependent transport systems inner membrane component precursor*
CTN_RS04720	-3,013	*Formate--tetrahydrofolate ligase*
CTN_RS04145	-3,021	*ATP synthase A chain*
CTN_RS02910	-3,033	*Alcohol dehydrogenase, iron-containing*
CTN_RS01985	-3,045	*Alpha-L-arabinofuranosidase*
CTN_RS07950	-3,045	*Hypothetical Protein*
CTN_RS03780	-3,048	*hypothetical protein*
CTN_RS00040	-3,053	*Putative uncharacterized protein*
CTN_RS04660	-3,065	*Phosphate butyryltransferase*
CTN_RS01205	-3,075	*Extracellular solute-binding protein family 1 precursor*
CTN_RS02900	-3,075	*XylU-related protein*
CTN_RS04330	-3,080	*Hypothetical Protein*
CTN_RS06125	-3,091	*Flagellar basal-body rod protein FlgC*
CTN_RS07960	-3,095	*Putative uncharacterized protein*
CTN_RS02895	-3,101	*Sugar ABC transporter, periplasmic sugar-binding protein*
CTN_RS01865	-3,107	*Beta-D-galactosidase*
CTN_RS01900	-3,116	*Alcohol dehydrogenase GroES domain protein*
CTN_RS04155	-3,123	*ATP synthase B chain*
CTN_RS07730	-3,136	*Aldehyde dehydrogenase*
CTN_RS09080	-3,203	*Putative uncharacterized protein*
CTN_RS02580	-3,235	*Putative uncharacterized protein*
CTN_RS07855	-3,252	*Transcriptional regulator, AraC family*
CTN_RS07945	-3,256	*Putative uncharacterized protein*
CTN_RS00490	-3,268	*Putative uncharacterized protein*
CTN_RS04055	-3,269	*ATP-dependent protease LA*
CTN_RS01695	-3,272	*oxidoreductase, short chain dehydrogenase/reductase family*
CTN_RS02685	-3,316	*Alkaline phosphatase precursor*
CTN_RS00240	-3,339	*Metal dependent phosphohydrolase*
CTN_RS07985	-3,351	*Putative uncharacterized protein*

CTN_RS00790	-3,358	*co-chaperone GroES*
CTN_RS02585	-3,367	*Acyl carrier protein*
CTN_RS05805	-3,371	*ComFC protein*
CTN_RS04160	-3,383	*F-ATPase delta-subunit*
CTN_RS00210	-3,408	*nucleotidyltransferase domain-containing protein*
CTN_RS04670	-3,422	*2-oxoisovalerate oxidoreductase, gamma subunit*
CTN_RS00165	-3,437	*dTDP-glucose 4,6-dehydratase*
CTN_RS04230	-3,443	*Putative uncharacterized protein*
CTN_RS04735	-3,448	*Exodeoxyribonuclease 7 small subunit*
CTN_RS03530	-3,519	*CRISPR-associated exonuclease, Cas4 family*
CTN_RS08660	-3,528	*S-layer-like array protein*
CTN_RS07805	-3,529	*Putative uncharacterized protein*
CTN_RS08120	-3,545	*Putative uncharacterized protein*
CTN_RS00055	-3,557	*Putative uncharacterized protein*
CTN_RS04710	-3,566	*Putative uncharacterized protein*
CTN_RS09090	-3,575	*Glycosyl transferase family 2*
CTN_RS05760	-3,604	*FOG WD40-like repeat-like protein*
CTN_RS03110	-3,649	*Binding-protein-dependent transport system inner membrane component*
CTN_RS06485	-3,650	*Sugar ABC transporter, ATP-binding protein*
CTN_RS00305	-3,672	*Sugar ABC transporter, permease protein*
CTN_RS05585	-3,677	*Flagellar L-ring protein precursor*
CTN_RS09365	-3,699	*Protein-L-isoaspartate O-methyltransferase*
CTN_RS04715	-3,702	*N utilization substance protein B like protein*
CTN_RS03665	-3,749	*Nicotinate-nucleotide pyrophosphorylase*
CTN_RS07700	-3,783	*Binding-protein-dependent transport systems inner membrane component*
CTN_RS00290	-3,826	*Uncharacterized FAD-dependent dehydrogenase-like protein*
CTN_RS00420	-3,870	*Crossover junction endodeoxyribonuclease ruvC*
CTN_RS02965	-3,871	*DNA ligase*
CTN_RS05580	-3,891	*Flagellar P-ring protein precursor*
CTN_RS08905	-3,896	*ABC transporter, ATP-binding protein*
CTN_RS07825	-3,928	*Putative uncharacterized protein*
CTN_RS07065	-3,932	*Diguanylate cyclase*
CTN_RS03520	-3,948	*CRISPR-associated helicase, Cas3 family*

CTN_RS05520	-3,978	*Transcriptional regulator, BadM/Rrf2 family*
CTN_RS08010	-3,997	*Putative uncharacterized protein*
CTN_RS07990	-4,019	*Putative uncharacterized protein*
CTN_RS09370	-4,061	*CinA-like protein*
CTN_RS01690	-4,121	*transcriptional regulator, RpiR family*
CTN_RS05795	-4,127	*Putative uncharacterized protein*
CTN_RS06685	-4,140	*Radical SAM domain protein*
CTN_RS04685	-4,144	*Pyruvate ferredoxin/flavodoxin oxidoreductase*
CTN_RS04150	-4,146	*ATP synthase C chain*
CTN_RS08640	-4,161	*Hemolysin-related protein*
CTN_RS02565	-4,187	*primosomal protein N*
CTN_RS07070	-4,189	*beta-lactamase domain protein*
CTN_RS08020	-4,203	*Integrase-recombinase protein*
CTN_RS09520	-4,208	*Putative uncharacterized protein*
CTN_RS04320	-4,218	*DNA-directed RNA polymerase subunit omega*
CTN_RS03040	-4,234	*Anti-sigma-28 factor, FlgM*
CTN_RS01180	-4,235	*Sugar binding protein of ABC transporter*
CTN_RS07045	-4,238	*Putative uncharacterized protein*
CTN_RS03195	-4,259	*Alpha-glucuronidase*
CTN_RS07875	-4,283	*Putative uncharacterized protein*
CTN_RS09535	-4,323	*M-related protein*
CTN_RS03050	-4,331	*Flagellar hook-associated protein 1*
CTN_RS03870	-4,361	*Alpha-mannosidase*
CTN_RS06840	-4,423	*Extracellular solute-binding protein family 1 precursor*
CTN_RS03170	-4,446	*Binding-protein-dependent transport systems inner membrane component*
CTN_RS01685	-4,500	*Phosphoglycerate dehydrogenase*
CTN_RS03175	-4,508	*Binding-protein-dependent transport systems inner membrane component*
CTN_RS08910	-4,556	*Putative uncharacterized protein*
CTN_RS01405	-4,556	*threonine ammonia-lyase*
CTN_RS02390	-4,565	*DNA integrity scanning protein disA*
CTN_RS04005	-4,577	*UDP-sugar hydrolase*
CTN_RS01700	-4,589	*TRAP dicarboxylate transporter, DctM subunit precursor*
CTN_RS03675	-4,604	*L-aspartate dehydrogenase*

CTN_RS01800	-4,656	*putative periplasmic binding protein*
CTN_RS01230	-4,717	*Sugar ABC transporter, permease protein*
CTN_RS05445	-4,757	*Flagellar biosynthesis-related protein*
CTN_RS01235	-4,814	*Sugar ABC transporter, permease protein*
CTN_RS05575	-4,855	*Putative uncharacterized protein*
CTN_RS02795	-4,883	*Beta-lactamase domain protein*
CTN_RS05755	-4,907	*tRNA pseudouridine synthase A*
CTN_RS00785	-4,907	*chaperonin GroL*
CTN_RS03670	-4,931	*Quinolinate synthetase A*
CTN_RS07905	-4,956	*Putative uncharacterized protein*
CTN_RS02500	-4,957	*ATPase*
CTN_RS07870	-5,008	*Transposase*
CTN_RS07725	-5,040	*Putative uncharacterized protein*
CTN_RS03525	-5,132	*CRISPR-associated helicase, Cas3 family*
CTN_RS03200	-5,138	*Putative uncharacterized protein*
CTN_RS06490	-5,149	*Prevent-host-death family protein*
CTN_RS07705	-5,161	*Binding-protein-dependent transport systems inner membrane component precursor*
CTN_RS08975	-5,213	*Putative uncharacterized protein*
CTN_RS08615	-5,231	*Protein grpE*
CTN_RS03875	-5,329	*Glycosidase*
CTN_RS08665	-5,361	*hypothetical protein*
CTN_RS03185	-5,394	*Oligopeptide ABC transporter, ATP-binding protein*
CTN_RS03880	-5,459	*Sugar ABC transporter, permease protein*
CTN_RS05870	-5,461	*Putative uncharacterized protein*
CTN_RS05495	-5,658	*Hypothetical Protein*
CTN_RS07710	-5,741	*Fructose-6-phosphate aldolase*
CTN_RS08610	-5,752	*Heat-inducible transcription repressor hrcA*
CTN_RS03510	-5,862	*CRISPR-associated autoregulator, Cst2 family*
CTN_RS01240	-5,898	*Sugar ABC transporter, periplasmic sugar-binding protein*
CTN_RS07720	-5,924	*Putative uncharacterized protein*
CTN_RS03505	-5,972	*Putative uncharacterized protein*
CTN_RS09650	-5,985	*Unknown*
CTN_RS03895	-6,025	*Transcriptional regulator, LacI family*

CTN_RS00160	-6,131	*Putative dTDP-6-deoxy-D-glucose-3,5-epimerase*
CTN_RS02800	-6,149	*FAD-dependent pyridine nucleotide-disulphide oxidoreductase*
CTN_RS03180	-6,160	*Oligopeptide ABC transporter, ATP-binding protein*
CTN_RS01270	-6,328	*ROK family protein*
CTN_RS00220	-6,338	*Uncharacterized protein*
CTN_RS05510	-6,361	*Endo-1,4-beta-glucanase E*
CTN_RS02805	-6,505	*Isochorismatase-related protein*
CTN_RS07715	-6,548	*Putative uncharacterized protein*
CTN_RS03190	-6,606	*Oligopeptide ABC transporter, periplasmic oligopeptide-binding protein*
CTN_RS03045	-6,648	*Putative uncharacterized protein*
CTN_RS05440	-6,659	*Putative uncharacterized protein*
CTN_RS06770	-7,069	*Transcriptional regulator, LacI family*
CTN_RS00615	-7,080	*Transposase, IS605 OrfB family*
CTN_RS09530	-7,087	*Putative uncharacterized protein*
CTN_RS01545	-7,107	*nitroreductase family protein*
CTN_RS03515	-7,221	*CRISPR-associated protein Cas5 family*
CTN_RS03890	-7,238	*Sugar ABC transporter, periplasmic sugar-binding protein*
CTN_RS03860	-7,246	*Putative uncharacterized protein*
CTN_RS03555	-7,306	*Xylose isomerase*
CTN_RS01710	-7,347	*ABC transporter, periplasmic substrate-binding protein*
CTN_RS03160	-7,399	*Endo-1,4-beta-xylanase A precursor*
CTN_RS09515	-7,460	*Flagellar protein*
CTN_RS08915	-7,606	*NADPH-dependent 7-cyano-7-deazaguanine reductase*
CTN_RS01220	-7,828	*Xylose isomerase domain protein TIM barrel*
CTN_RS08130	-8,074	*Putative uncharacterized protein*
CTN_RS01500	-8,385	*Heat shock protein Hsp20*
CTN_RS03115	-8,430	*Periplasmic binding protein*
CTN_RS09525	-8,513	*Basal-body rod modification protein FlgD*
CTN_RS01705	-8,819	*Tripartite ATP-independent periplasmic transporter, DctQ component*
CTN_RS06755	-8,977	*Binding-protein-dependent transport systems inner membrane component*
CTN_RS01225	-9,049	*Sugar ABC transporter, ATP-binding protein*
CTN_RS03165	-9,106	*Transposase, IS605 OrfB family*
CTN_RS05505	-9,130	*Endo-1,4-beta-glucanase A*

CTN_RS06765	-9,222	*Oligopeptide ABC transporter, ATP-binding protein*
CTN_RS03885	-9,229	*Sugar ABC transporter, permease protein*
CTN_RS06760	-10,030	*Oligopeptide/dipeptide ABC transporter, ATPase subunit*
CTN_RS05850	-10,396	*hypothetical protein*
CTN_RS02810	-10,569	*Flagellin*
CTN_RS01475	-11,314	*Transcriptional regulators-like protein*
CTN_RS08495	-11,780	*ATPase AAA-2 domain protein*
CTN_RS01870	-13,438	*peptide ABC transporter substrate-binding protein*
CTN_RS01480	-15,628	*Hypothetical Protein*
CTN_RS01265	-16,089	*Alcohol dehydrogenase, zinc-containing*
CTN_RS06750	-16,147	*Binding-protein-dependent transport systems inner membrane component*
CTN_RS01495	-16,919	*Chaperone protein dnaK*
CTN_RS01245	-17,329	*Xylose isomerase domain protein TIM barrel*
CTN_RS01260	-20,209	*Creatinine amidohydrolase*
CTN_RS01250	-22,618	*PfkB domain protein*
CTN_RS01255	-22,765	*Oxidoreductase domain protein*
CTN_RS06730	-53,855	*Beta-mannosidase*
CTN_RS06735	-82,574	*Glycosidase*
CTN_RS06740	-91,573	*ROK family protein*
CTN_RS06745	-107,213	*Oligopeptide ABC transporter, periplasmic oligopeptide-binding protein*
