# Supplementary material for: CO2-Induced Transcriptional Reorganization: Molecular Basis of Capnophillic Lactic Fermentation in Thermotoga neapolitana
Source: Front Microbiol. 2020 Feb 18;11:171. doi: 10.3389/fmicb.2020.00171 (PMC7039931; doi:10.3389/fmicb.2020.00171)
Supplement: Supplementary file 2 [file Data_Sheet_2.docx]

Supplementary Material

**Supplemental Material Figure SM1** – GO enrichment analysis of (**A**) up-regulated DEGs and (**B**) down-regulated DEGs by RNA-seq approach. Bars indicate the percentage of genes related to GO categories from DEGs list (Black bars) and from Genome list (Grey bars).


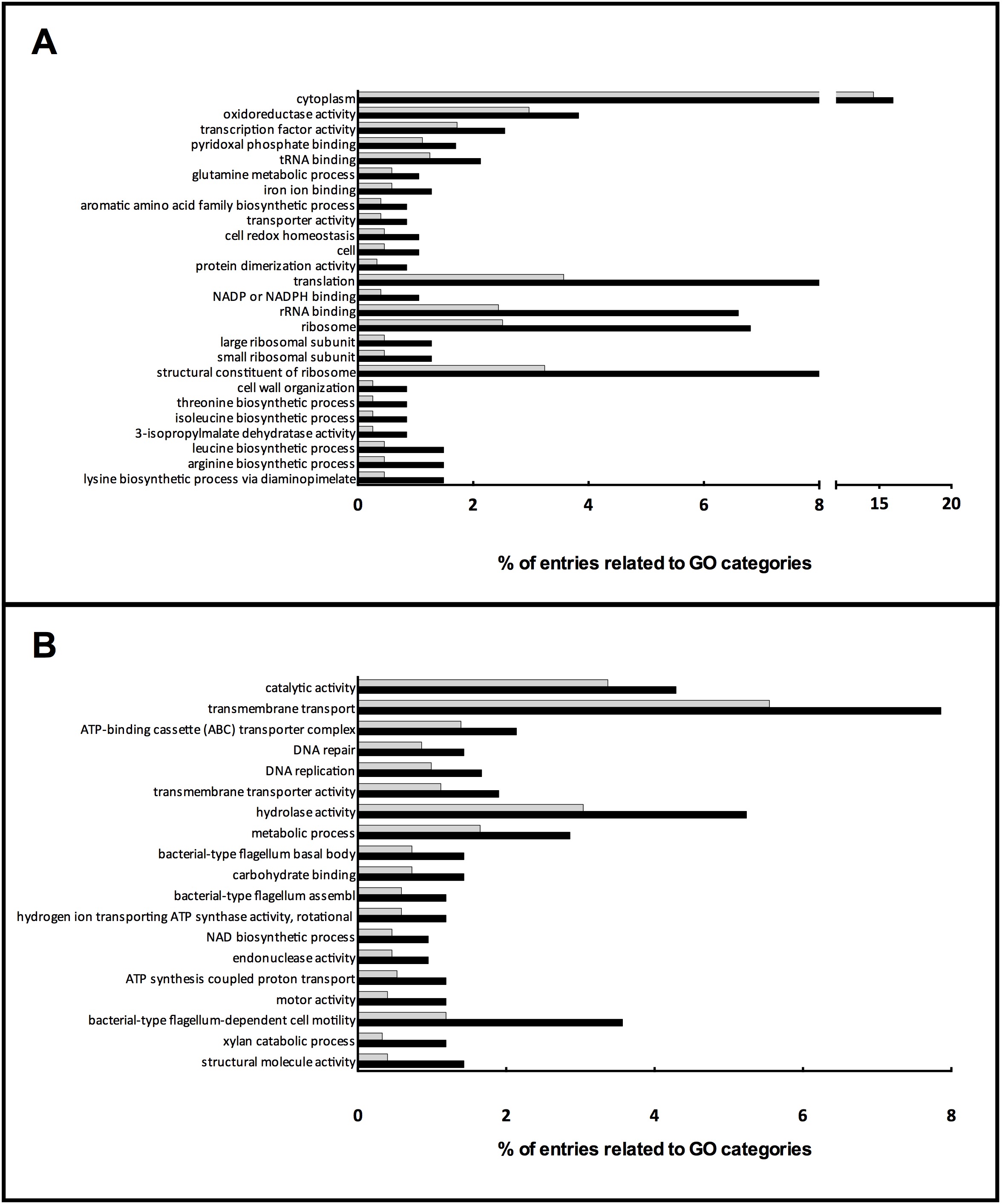


**Supplemental Material Figure SM2** Validation of RNAseq results by real-time PCR. The selected genes were CTN_RS03380 (pFORa), CTN_RS05285 (HYDa), CTN_RS03950 (LDH), CTN_RS02345 (PFK) and CTN_RS00550 (EDD). Real time PCR variations are indicated as significant (p.value ≤ 0.05) fold-change in CO_2_ (grey bars) with respect to control (N_2_ – black bars). mRNA levels were calculated relatively to the expression of the RNA 16S used as calibrator. Correspondent RNA-seq expression results were showed as normalized Fold change value ( see Material & Methods) at FDR ≤ 0.05.

**
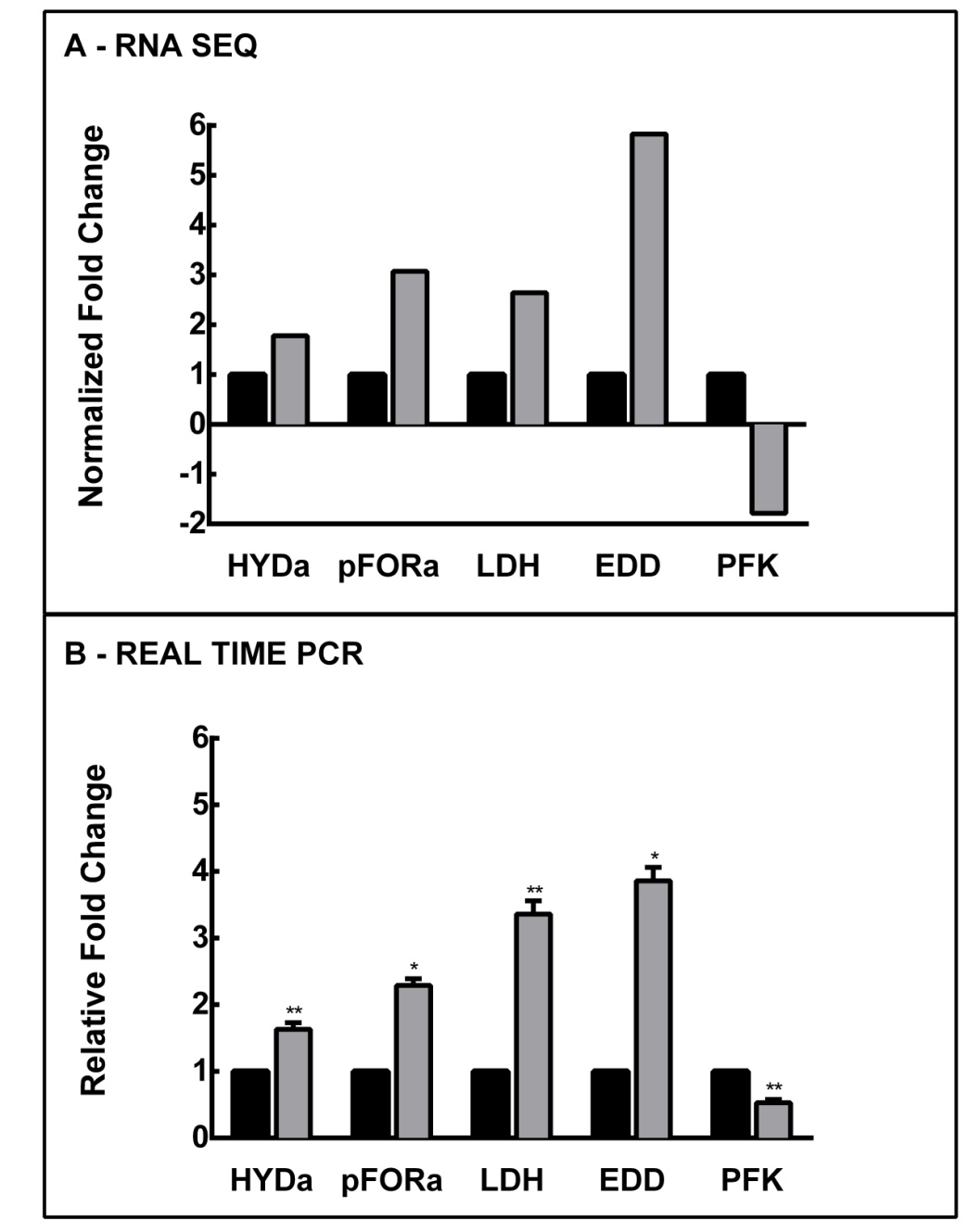
**

**Supplemental Material Figure SM3** – A) DEGs clustering analysis CLF conditions.The analysis was obtained by a k-mean approach. B) Descriptions of clustered DEGs .

**
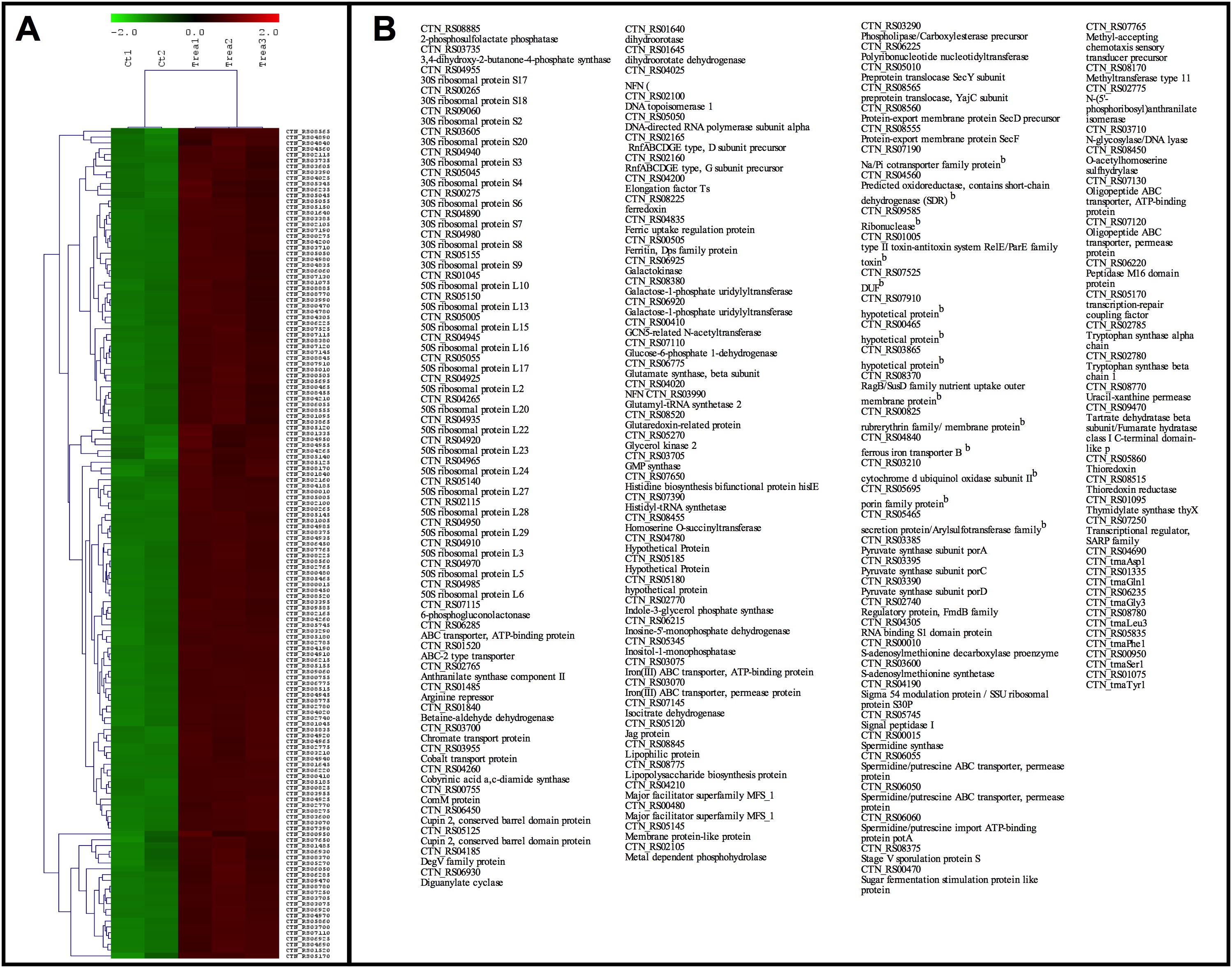
**

**Supplemental Material Figure SM4 –**Electron microscope images of cells treated with N_2_ (top) and CO_2_ (bottom) at 48h.


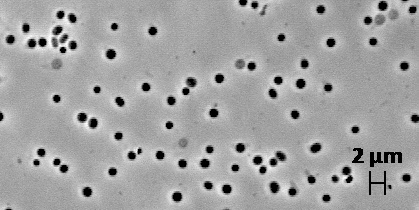


**
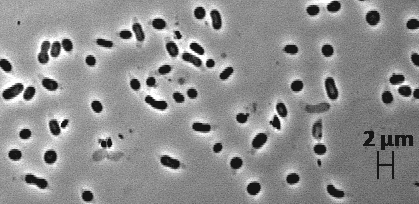
**

**Supplemental material Table SM1** - Rna-seq allignment stats

| **Treatment** | **Replicate** | **HQ sequenced reads** | **Mapped reads (%)** | **Unmapped reads (%)** |
| --- | --- | --- | --- | --- |
| **N_2_** | 1 | 19390095,00 | 98,92 | 1,08 |
| **N_2_** | 2 | 2100912,00 | 98,59 | 1,41 |
| **N_2_** | 3 | 24732148,00 | 98,76 | 1,24 |
| **CO_2_** | 1 | 28835603,00 | 99,24 | 0,76 |
| **CO_2_** | 2 | 31800340,00 | 99,06 | 0,94 |
| **CO_2_** | 3 | 29573417,00 | 99,05 | 0,95 |

**Supplemental material Table SM2** – List of primers used for Real Time PCR.

| **Locus** | **Name** | **Sequence** |
| --- | --- | --- |
| CTN_RS03380 | pFORa FW | CCACAAAGTTGGCGCATTGA |
|  | pFORa RV | GTCAAGGACGATGACGCTCT |
| CTN_RS05285 | HYDa FW | CGCAGAACACAACTATCCAC |
|  | HYDa RV | TCTCCTTCATGCCGTGTACT |
| CTN_RS03950 | LDH FW | CTGCAACAGGTGCGATTCTC |
|  | LDH RV | AGCGTAATGTGTTGCTCCCT |
| CTN_RS02345 | PFK FW | AGGCTACGAGACGAGGATCA |
|  | PFK RV | GGCTTCTACTCCCATGCTCA |
| CTN_RS00550 | EDD FW | TAGAGAGGGTGGGCTTGGAA |
|  | EDD Rv | TGCTTCATCTTTTCGGGGACA |
| CTN_rRNA16S | 16S FW | TACCCCATACGCTCCATCAA |
|  | 16S Rv | GTAGTTGGTGGGGTAACGG |
